# Nanobody regulation of C-type inactivation in Kv1.3 channels

**DOI:** 10.1038/s41467-026-74630-8

**Published:** 2026-08-04

**Authors:** Purushotham Selvakumar, Kenton J. Swartz, Ana I. Fernández-Mariño

**Affiliations:** 1 Molecular Physiology and Biophysics Section, Porter Neuroscience Research Center, National Institute of Neurological Disorders and Stroke, National Institutes of Health, Bethesda, MD USA; 2 Department of Physiology and Biophysics, University of Colorado, Anschutz Medical Campus, Aurora, CO USA

**Keywords:** Permeation and transport, Cryoelectron microscopy, Immunotherapy, Electron microscopy

## Abstract

Nanobodies are powerful tools for modulating ion channels for mechanistic investigations and developing new therapeutics. The Kv1.3 channel is highly expressed in T-lymphocytes where it promotes sustained T-cell activation, its expression is elevated in autoimmune disorders and inhibitory nanobodies are immunosuppressive. The A019400G09 nanobody (NB1.3) binds to the external surface of Kv1.3 and inhibits the channel by promoting slow C-type inactivation of the ion selectivity filter. Here we explore the mechanism by which NB1.3 promotes inactivation by determining a series of cryo-EM structures of Kv1.3 and mutating the interface between NB1.3 and the channel. Our results reveal that interaction of NB1.3 with both the S1-S4 voltage-sensing domain and the turret within the pore domain are required to promote inactivation. We also identify a network of interacting hydrophobic residues linking the turret to the ion selectivity filter that stabilizes the conducting state and mediate the actions of NB1.3. These findings provide a foundation for developing therapeutics targeting Kv1.3 channels and exploring how nanobodies can interact with other tetrameric cation channels to modulate their activity.

## Abstract (web summary)

Here, authors use cryo-EM, electrophysiology and mutagenesis to uncover how a nanobody promotes C-type inactivation of the T-cell Kv1.3 potassium channel by disrupting a conserved hydrophobic network linking the turret region to the selectivity filter.

## Introduction

Nanobodies are single domain antibodies produced by camelids that are powerful tools for studying key mechanistic features of membrane proteins, and they are emerging as valuable therapeutics^1–3^. The similarity of nanobodies to the variable domain of human immunoglobulins reduces their immunogenicity, enhancing their utility for use in humans as therapeutics. The specificity of nanobodies for their desired targets are comparable to conventional antibodies, but their smaller size enables them to reach smaller or less accessible epitopes, in some cases enabling the nanobody to stabilize specific functional states. For example, a nanobody generated against the β2-adrenoceptor was critical for solving the structure of the elusive active state^4^, and more recently, a structure of the A019400G09 nanobody (NB1.3) inhibitor bound to the inactivated state of human Kv1.3 (hKv1.3) voltage-activated potassium (Kv) channel^5,6^ provided a first glimpse for how nanobodies interact with tetrameric cation channels.

The Kv1.3 channel that NB1.3 targets is abundantly expressed in T-lymphocytes where it plays an essential role in regulating immune responses^7–10^. T cell activation is initiated when the T cell receptor engages with an antigen-presenting cell, triggering the rapid influx of Ca^2+^. Entry of positively charged Ca^2+^ ions depolarizes the T cell membrane, which leads to opening of Kv1.3 and repolarization of the membrane to maintain the necessary driving force for the sustained Ca^2+^ entry required for T cell activation and proliferation. Inhibitors of Kv1.3 have therefore been sought after to modulate T cell activation and suppress immune responses^11–18^. Indeed, Kv1.3 inhibitors are being developed for treating autoimmune diseases, including multiple sclerosis, type 1 diabetes, psoriasis, graft-versus-host disease and rheumatoid arthritis^19–22^.

**Fig. 1:**
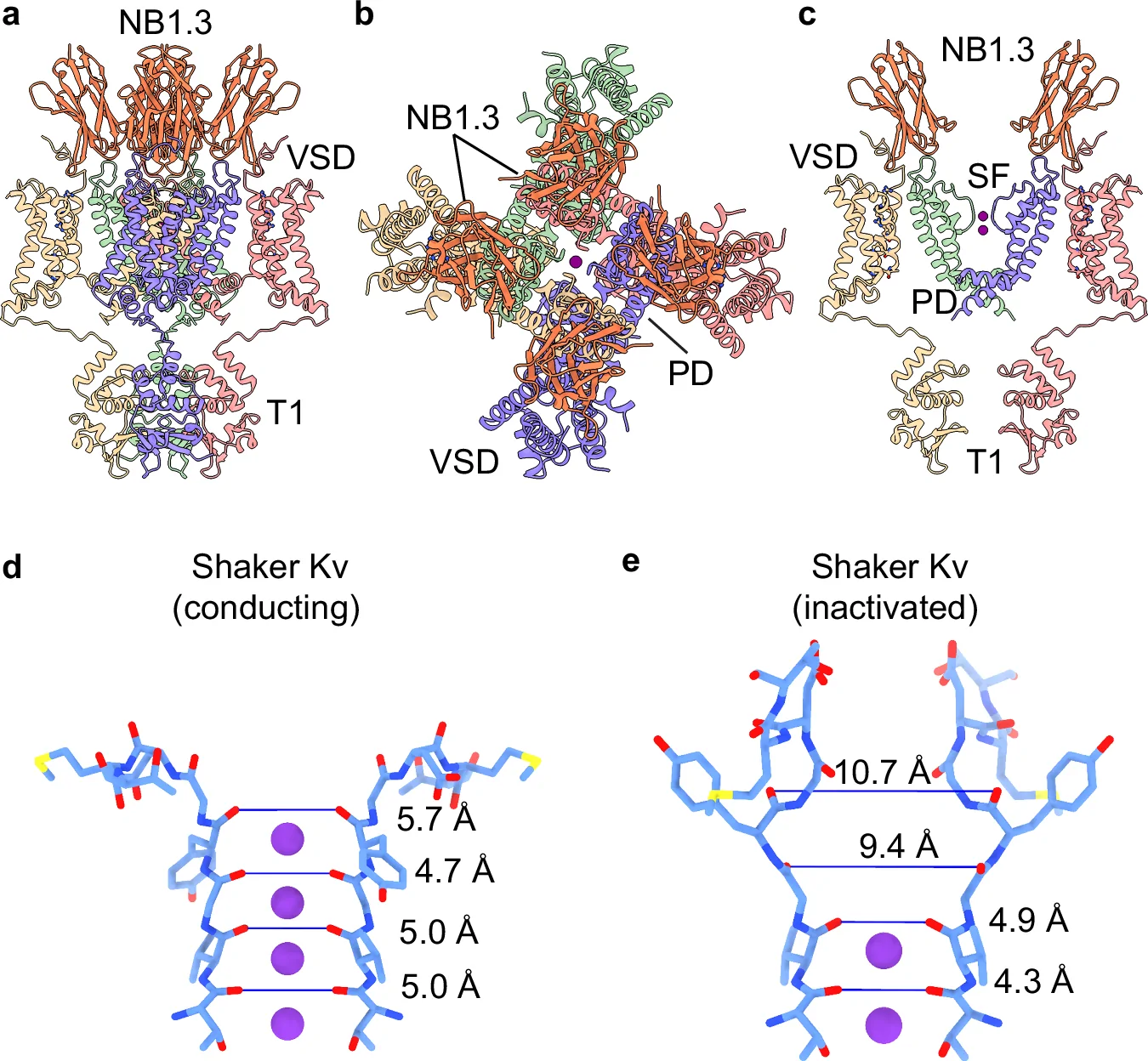
Structure of hKv1.3 with NB bound and structures of the ion selectivity filter of the Shaker Kv channel in conducting and C-type inactivated conformations. **a**, **b** Side and external views of the structure of hKv1.3 with four NB1.3 nanobodies bound to the external side of the channel and interacting with both the pore domain (PD) and voltage-sensing domains (VSD). Cytoplasmic tetramerization domain (T1). **c** Cutaway side view of the hKv1.3 model bound to NB1.3, showing interactions with both the VSD and PD. Ion selectivity filter (SF). Front and rear S1–S4 domains and contributions to the T1 domain are omitted for clarity. **d** Structural model of the ion selectivity filter of the Shaker Kv channel in a conducting conformation (pdb id: 7SIP). **e** Structural model of the ion selectivity filter of the Shaker Kv channel in a dilated inactivated conformation (pdb id: 8TEO).

The Kv1.3 channel that is inhibited by NB1.3 is one of eight members of the mammalian Kv1 family and the only one expressed in human T cells^7,9,10,23,24^. Kv1 channels are tetrameric cation channels where each subunit containing six transmembrane (TM) helices, with the S1–S4 helices forming each voltage-sensing domain (VSD) and the S5–S6 helices forming the central ion conducting pore domain (Fig. 1a–c)^5,25–29^. Kv1 channels are also domain-swapped tetramers, with the S1–S4 domain of one subunit positioned nearby the S5–S6 helices of the adjacent subunit (Fig. 1a–c). The S5–S6 pore domain contains many critical structural elements at the external end of the pore; the turret immediately after the S5 helix, followed by the reentrant pore helix and the P-loop that forms the ion selectivity filter (Fig. 1c), where backbone carbonyl groups coordinate K^+^ ions at four sites required for rapid and highly selective K^+^ permeation (Fig. 1d)^5,25–31^. In the continual presence of the activating depolarizing stimulus, Kv1 channels undergo slow C-type inactivation^32–34^ caused by a localized conformational change in the ion selectivity filter that dilates the filter, resulting in the loss of the two outermost K^+^ ion binding sites required for rapid ion permeation (Fig. 1d, e)^5,29,35–38^. Functional studies demonstrate that NB1.3 inhibits the hKv1.3 channel by promoting C-type inactivation^5,6^, and a recent cryo-EM structure of NB1.3 bound to hKv1.3 revealed that four NBs binds to the extracellular surface of the channel^5^, interacting with the S1–S4 voltage-sensing domain of one subunit and the turret within the pore domain of the neighboring subunit (Fig. 1a, b). However, the mechanism by which NB1.3 promotes inactivation remains unclear because the structure of the apo hKv1.3 channel in the absence of NB1.3 also adopted a C-type inactivated conformation with a dilated ion selectivity filter^5^.

In the present study, we investigated the mechanism by which NB1.3 promotes inactivation by determining a series of cryo-EM structures of the hKv1.3 channel in conducting and inactivated states with and without NB1.3 bound. Mutagenesis at the interface between NB1.3 and both the S1–S4 domain and turret demonstrate that both interfaces are required for NB1.3 to promote inactivation and identify the turret as a hot spot for tuning slow inactivation. Comparison of several new and previously available cryo-EM structures lead to the identification of a hydrophobic network spanning from a conserved Phe in the turret to the ion selectivity filter that is disrupted as the channel inactivates. Taken together, our findings identify an important network between the turret and ion selectivity filter that is engaged by NB1.3 to regulate slow inactivation.

## Results

### Structure of hKv1.3 with the ion selectivity filter in a conducting conformation

**Fig. 2:**
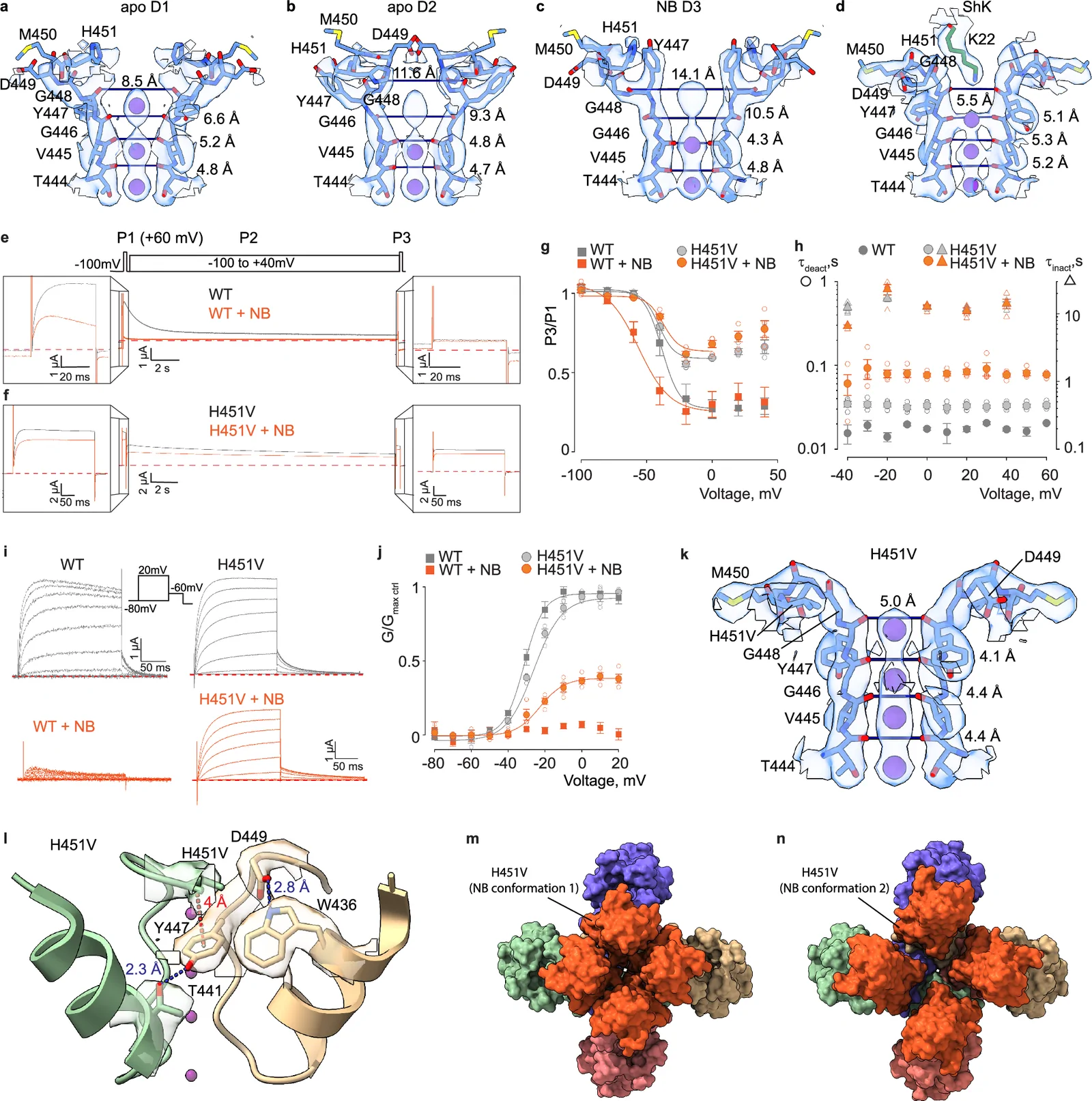
Structure of hKv1.3 with C-type inactivated and conducting ion selectivity filters. **a**, **b** Cryo-EM density and structural models of the ion selectivity filter of apo hKv1.3 in D1 and D2 conformations. **c** Cryo-EM density and structural model of the ion selectivity filter of hKv1.3 with NB1.3 bound in the D3 conformation. **d** Cryo-EM density and structural model of the ion selectivity filter of hKv1.3 with ShK-Fab bound in a presumably conducting conformation. **e**, **f** Current traces obtained using a three-pulse protocol without leak subtraction for WT hKv1.3 and the H451V mutant in the absence and presence of NB1.3. **g** Plot of *P*3/*P*1 for WT hKv1.3 and the H451V mutant in the absence and presence of 100 nM NB1.3. Smooth curves are Boltzmann fits with *V*_1/2_ and *z* values of −40.8 mV and 4.4 for H451V in control solution, −37.4 mV and 7.1 for H451V in the presence of NB1.3 (*n* = 4 in 3 independent experiments), −38.4 mV and 3.8 for WT in control solution and −56.1 mV (*n* = 9 in 7 independent experiments) and 2.2 for WT in the presence of NB1.3 (*n* = 5 in 4 independent experiments). Error bars represent S.E.M. **h** Plot of time constants (*τ*) for inactivation (light gray and orange triangles) or deactivation (light gray and orange circles) for H451V in the absence or presence of 100 nM NB1.3. A single exponential function was fit to the inactivation of H451V using current traces elicited during P2, as illustrated in (**f**). Deactivation *τ* values were obtained from single exponential functions fit to the tail currents illustrated in (**i**). For WT hKv1.3 (dark gray circles) *n* = 3 in 3 independent experiments. For H451V, *n* = 4 in 3 independent experiments. Error bars represent S.E.M. **i** Families of currents elicited by depolarization to voltages between −80 and +20 in the absence and presence of 100 nM NB1.3 for WT hKv1.3 and the H451V mutant. Holding voltage was −80 mV, and tail voltage was −60 mV. **j** Plot of normalized conductance (*G*/*G*_max_) obtained from tail current measurements for WT hKv1.3 and the H451V mutant in the absence and presence of NB1.3. Smooth curves are Boltzmann fits with *V*_1/2_ and *z* values of −27.4 mV and 4.4 for H451V in control solution, −22.7 mV and 4.0 for H451V in the presence of NB1.3 (*n* = 4 in 3 independent experiments), −31.3 mV and 4.8 for WT in control solution (*n* = 4 in 3 independent experiments) and for WT in the presence of NB1.3 currents were too small for reliable fitting (*n* = 3 in 3 independent experiments). Error bars represent S.E.M. **k** Cryo-EM density and structural model of the ion selectivity filter of the H451V mutant of hKv1.3. **l** Cryo-EM density and structural model of the ion selectivity filter of the H451V mutant of hKv1.3 showing side chain interactions behind the selectivity filter. Hydrogen bonds are indicated by blue dotted lines and hydrophobic interactions by red dotted lines. **m** Surface representation of a structural model of the external surface of hKv1.3 with NB1.3 bound in conformation 1 (inward). **n** Surface representation of a structural model of the external surface of hKv1.3 with NB1.3 bound in conformation 2 (outward).

Highly K^+^ selective ion channels contain an ion selectivity filter at the external end of the pore where K^+^ ions are coordinated by backbone carbonyl groups at four sites (S1–S4) strategically positioned to replace waters of hydration to achieve exquisite K^+^ selectivity, with multioccupancy of the filter enabling high rates of ion permeation (Fig. 1d)^30,31^. The selectivity filters in our earlier structures of apo hKv1.3 and with NB1.3 bound in 150 mM K^+^ exhibited varying extents of dilation (D1–D3; Fig. 2a–c) even though such high K^+^ concentrations are sufficient to stabilize a conducting state of the related Shaker Kv channel^29,32,35^, suggesting that the hKv1.3 channel is particularly prone to inactivate. In apo hKv1.3, D1 and D2 conformations were observed, whereas with NB1.3 bound, D1 and D3 conformations were observed (Fig. 2a–c)^5^. In each of these conformations, density corresponding to K^+^ ions bound can be observed at two sites within the inner half of the filter, whereas the variable dilation of backbone carbonyl groups in the outer half of the filter would be expected to diminish K^+^ occupancy (Fig. 2a–c). Although density possibly corresponding to one K^+^ can be observed in the outer half of the filter in D1, D2 and D3 conformations, the position of backbone carbonyl groups would be more consistent with this density corresponding to water. Indeed, structures of the C-type inactivated state of the related Shaker Kv channel are similar to the D2 conformation of hKv1.3 (Fig. 1e), and molecular dynamics (MD) simulations suggest that density in the outer half of the filter corresponds to water^29,35^. In contrast, a structure of hKv1.3 bound by the ShK sea anemone toxin coupled to a Fab fragment adopt a more conventional conducting conformation even though a Lys residue from the toxin inserts into the outer end of the filter to diminish K^+^ occupancy at the S1 site (Fig. 2d)^5^. While functional experiments demonstrate that NB1.3 inhibits hKv1.3 by promoting slow C-type inactivation^5^, both increasing the rate of inactivation and shifting the steady-state inactivation relationship to more negative voltages (Fig. 2e, g, h), how NB1.3 binding promotes inactivation remained unclear because the available structures of hKv1.3 were either inactivated or blocked by ShK.

To provide a foundation for understanding the mechanism by which NB1.3 binding to hKv1.3 promotes C-type inactivation, we set out to solve a structure of the channel with the ion selectivity in a conducting state. The T449 position in the selectivity filter of Shaker strongly influences C-type inactivation, with polar substitutions speeding inactivation and hydrophobic substitutions, such as T449V slowing inactivation^32^. The equivalent position in hKv1.3 is a His (H451 in our construct; Fig. S1), and mutation of this His has been reported to slow inactivation^39^. We also found that the H451V mutation greatly slowed inactivation of hKv1.3 and prevented 100 nM NB1.3 from accelerating inactivation and shifting the stead-state inactivation relation to more negative voltages when assessed with long test depolarizations (Fig. 2e–h). Application of 100 nM NB1.3 to hKv1.3-H451V produced a detectable inhibition of Kv channel currents when elicited with shorter duration pulses, but this inhibitory effect was much smaller than observed with WT hKv1.3 (Fig. 2i, j) and did not increase when testing the NB at 1 µM (Fig. S2a, d). Although the mechanism for this inhibitory effect of NB1.3 for hKv1.3-H451V is unclear, aspects of channel gating other than inactivation are also affected by NB1.3, including a detectable slowing of deactivation in the hKv1.3-H451V mutant (Fig. 2h, i). The H451V mutant also rescued ion conduction in the W436F mutant of hKv1.3 (Fig. S2e), a substitution that in both Shaker (W434F) and Kv1.3 renders the channels nonconducting by promoting slow inactivation^29,40–44^, further supporting an influence of H451V on slow inactivation in hKv1.3.

We proceeded to express and purify the full-length hKv1.3-H451V channel and determined the cryo-EM structure with an overall resolution of 2.97 Å in 150 mM K^+^ concentration while imposing C4 symmetry (Table S1 and Fig. S3). The overall architecture of hKv1.3-H451V was similar to apo hKv1.3 solved previously^5^, with the S1–S4 voltage-sensing domain in an “up” or activated position, and the inner S6 gate open. In contrast, the selectivity filter adopted a conformation (Fig. 2k) very similar to that seen in other K^+^ selective channels thought to represent conducting conformations^25,29–31^, with backbone carbonyl groups in the filter positioned to coordinate K^+^ ions and density evident for K^+^ ions at all four sites in the filter (Figs. 1d and 2k). In addition, key hydrogen bonds known to stabilize conducting conformations of the selectivity filter in K^+^ channels are intact in hKv1.3-H451V, including the interaction of W436 with D449 and that of Y447 with T441 (Fig. 2l)^29,40^. The Val introduced at 451 is positioned to stack with Y447, with C-centroid distance of 4 Å between the side chain methyl group (CG2) and aromatic Y447 ring, making a CH–Π interaction that stabilizes a conducting conformation of the selectivity filter (Fig. 2l). We therefore conclude that the selectivity filter in the hKv1.3-H451V mutant is in a conducting conformation.

**Fig. 3:**
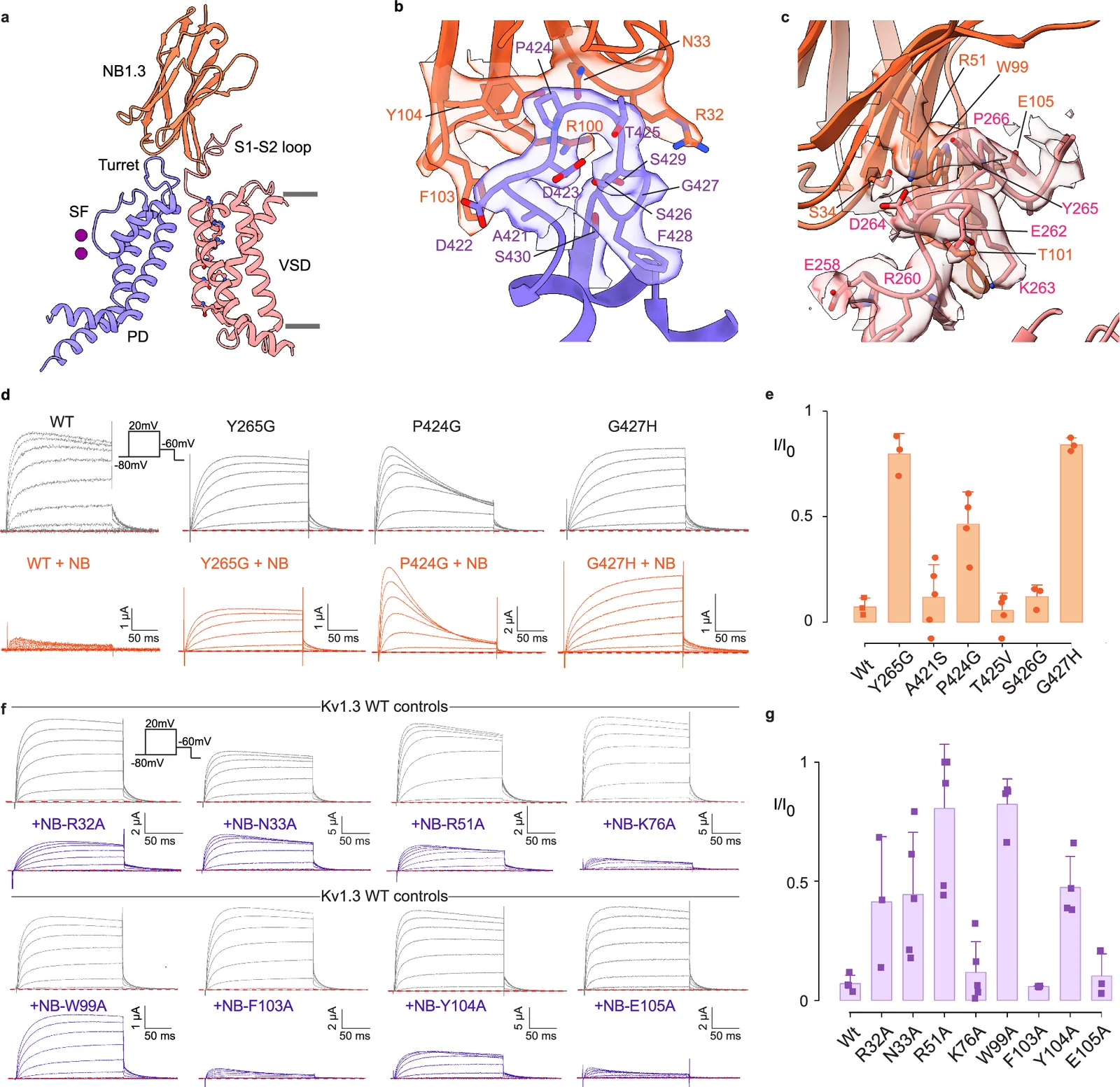
Interaction of NB1.3 with the S1–S4 voltage-sensing domain and turret of hKv1.3. **a** Structural model of hKv1.3 illustrating NB1.3 interacting with the S1–S2 loop within the VSD and the turret within the PD. **b** Cryo-EM density and structural models of the interaction of NB1.3 (orange) with the turret loop of hKv1.3 (purple). **c** Cryo-EM density and structural models of the interaction of NB1.3 (orange) with the S1–S4 domain of hKv1.3 (pink). **d** Families of currents elicited by depolarization to voltages between −80 and +20 mV in the absence and presence of 100 nM NB1.3 for WT hKv1.3 and the Y265G, P424G and G427H mutants. Holding voltage was −80 mV, and tail voltage was −60 mV. **e** Plot of normalized currents at 0 mV (*I*/*I*_0_) for WT hKv1.3 and different hKv1.3 mutants for 100 nM NB1.3 obtained from (**d**). Values for *n* and independent experiments were: 3 and 3 for WT, 3 and 3 for Y265G, 5 and 5 for A421S, 4 and 4 for P424G, 5 and 5 for T425V, 3 and 3 for S426G and 3 and 3 for G427H. Error bars represent S.E.M. **f** Families of currents elicited by depolarization to voltages between −80 and +20 mV of different NB1.3 mutants. Holding voltage was −80 mV and tail voltage was −60 mV. **g** Plot of normalized currents at 0 mV (*I*/*I*_0_) for WT hKv1.3 and different mutants of NB1.3 tested at 100 nM obtained from (**f**). Values for *n* and independent experiments were: 3 and 3 for WT, 3 and 2 for R32A, 5 and 3 for N33A, 6 and 5 for R51A, 5 and 4 for K76A, 4 and 3 for W99A, 3 and 2 for F103A, 4 and 3 for Y104A and 3 and 2 for E105A. Error bars represent S.E.M.

Having obtained a structure of apo hKv1.3 with the filter in a conducting conformation, we set out to solve a structure of the H451V mutant with NB1.3 bound to look for clues about how the NB might promote inactivation. Cryo-EM images of hKv1.3-H451V obtained in the presence of NB1.3 were initially processed using C1 symmetry, where four copies of the NB were observed to decorate the extracellular surface of the protein. From the ab initio reconstruction and heterogenous refinement, we observed 2 classes which differed only in the way the NB orients on extracellular surface of hKv1.3-H451V. In conformation 1 the NB1.3 leans inward towards the central axis of the pore and in conformation 2 the NB1.3 leans outward (Fig. 2m, n), similar to that in our earlier structure with NB1.3 bound to hKv1.3^5^. We then processed the data using C4 symmetry, and the density maps were refined to a final overall resolution of 3.06 Å for inward and 3.03 Å for outward conformations (Fig. 2m, n, Table S1, and Fig. S4). Both conformations were similar to the structure of NB1.3 bound to WT hKv1.3 with regard to the binding interfaces (Fig. S9), but in both conformations, the selectivity filter adopted a conducting conformation, indicating that stabilization of the conducting state by H451V was sufficient to preclude inactivation even with NB1.3 bound. In the case of the inward-leaning conformation 1, the only interaction between adjacent NBs is through intermolecular hydrogen bonds between K76 and S71 (Fig. S4k), which cannot form when the NB leans outward in conformation 2 (Fig. 2m, n). These two conformations of NB1.3 do not seem to be related to the mechanism by which the NB promotes inactivation as both contain selectivity filters in conducting conformations, and the K76A NB1.3 mutant stabilizing the inward leaning conformation has activity that is similar to WT NB1.3 (Fig. 3f, g).

### The S1–S2 loop and turret regions are critical for the actions of NB1.3

The structures of NB1.3 bound to hKv1.3 reveal that the NB interacts intimately with S1–S2 loop of hKv1.3 and with the turret region within the pore domain (Fig. 3a–c). Nine residues in the S1–S2 loop were resolved with NB1.3 bound (Fig. 3c), none of which were resolved in the apo hKv1.3 structure^5^. Of these, Y265 stood out because it interacts intimately with W99 in NB1.3 and is a Gly in the Kv1.6 and Kv1.7 channels that are not sensitive to the NB^5^. To investigate whether the interaction between Y265 and NB1.3 is critical, we made the Y265G mutation in hKv1.3 and found that although voltage-dependent activation and inactivation are not detectably altered (Fig. S5a–d), the inhibitory effect of 100 nM NB1.3 is greatly reduced and the NB no longer speeds inactivation (Fig. 3d, e). The modest inhibition that remains was not increased by raising the NB concentration at 1 µM (Fig. S2b, d), suggesting that the NB concentration remains saturating. We also mutated several residues on NB1.3 located near to Y265G at the interface with hKv1.3 and found that both W99A and R51A greatly reduced the inhibitory effects of NB1.3, and the NB no longer hastened inactivation (Fig. 3f, g), similar to the effects of the Y265G mutant in hKv1.3. In contrast, the E105A mutant of NB1.3 that is positioned near the S1–S2 loop but that doesn’t participate in specific interactions with that loop, exhibited activity similar to WT NB1.3 (Fig. 3f, g). Taken together, these findings indicate that interactions of NB1.3 with the S1–S2 extracellular loop within the S1–S4 voltage-sensing domains are critical for the modulatory actions of the NB on the hKv1.3 channel.

**Fig. 4:**
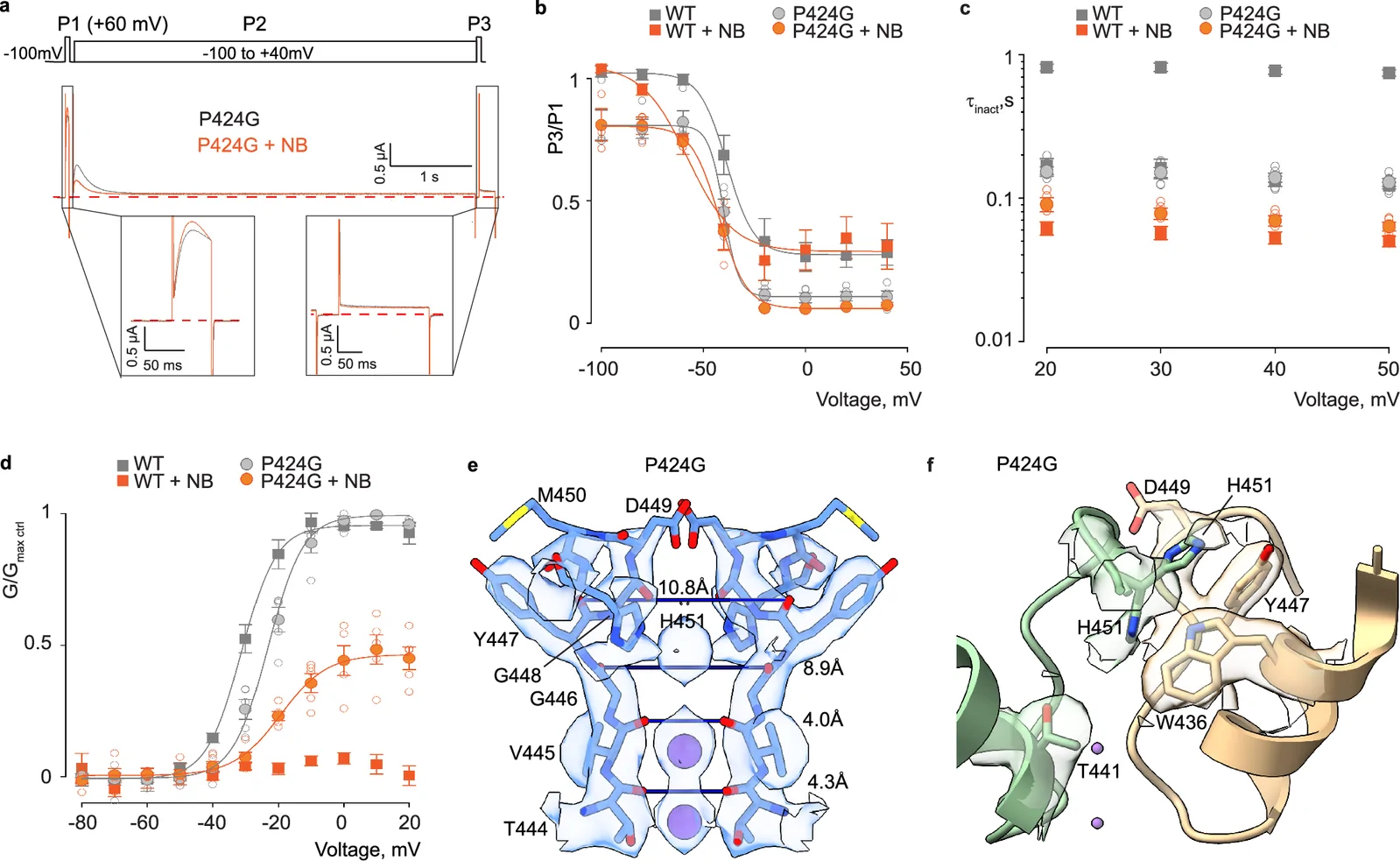
Functional characterization and structure of the P424G mutant of hKv1.3. **a** Current traces obtained using a three-pulse protocol without leak subtraction from a holding voltage of −100 mV for the hKv1.3 P424G mutant in the absence and presence of 100 nM NB1.3. **b** Plot of *P*3/*P*1 for WT hKv1.3 and the P424G mutant in the absence and presence of 100 nM NB1.3. Smooth curves are Boltzmann fits with *V*_1/2_ and *z* values of −40 mV and 7 for P424G in control solution (*n* = 4 in 3 independent experiments) and −42.3 mV and 4 in the presence of NB1.3 (*n* = 3 in 3 independent experiments). Error bars represent S.E.M. *P*3/*P*1 is less than 1 at negative P2 voltages due to incomplete recovery from inactivation (see “Methods”). WT data are from Fig. 1g. **c** Inactivation kinetics of WT hKv1.3 and the P424G mutant in the presence and absence of 100 nM NB1.3. *τ* values for WT were obtained by fitting a double exponential function in control to current traces in P2 and a single exponential function in the presence of NB1.3. For P424G a single exponential function was fit to current traces elicited during P2 in both control (*n* = 5 in 4 independent experiments) and in the presence of NB1.3 (*n* = 4 in 4 independent experiments) and WT are from^5^. Error bars represent S.E.M. **d** Plot of normalized conductance (*G*/*G*_max_) obtained from tail current measurements for WT hKv1.3 and the P424G mutant in the absence and presence of 100 nM NB1.3. Smooth curves are Boltzmann fits with *V*_1/2_ and *z* values of −23.2 mV and 4.8 for P424G in the control solution and −19.4 mV and 3.7 in the presence of NB1.3 (*n* = 5 in 4 independent experiments). Error bars represent S.E.M. WT data are from Fig. 1j. **e** Cryo-EM density and structural models of the dilated ion selectivity filter of the P424G mutant of hKv1.3 in a D2 conformation. **f** Cryo-EM density and structural model of the ion selectivity filter of the P424G mutant of hKv1.3 showing disruption of side chain interactions behind the selectivity filter and alternate H451 conformations.

Next, we explored the influence of mutations in the turret region with the pore domain, a region implicated in controlling C-type inactivation in the related Shaker Kv channel^29,45–47^. Guided by the cryo-EM structure of NB1.3 bound to hKv1.3 (Fig. 3a, b), we mutated residues in the turret loop positioned near to where NB1.3 binds. Mutations at A421, T425, and S426 exhibited voltage-dependent activation and inactivation that were similar to the WT hKv1.3 channel and the effects of NB1.3 were not dramatically altered (Fig. S5e–p). In contrast, mutation of G427 to His, the residue that occupies this position in most other Kv1 channels (Fig. S1), dramatically reduced the inhibition and the speeding of inactivation produced by 100 nM NB1.3 (Fig. 3d, e), implicating the turret in tuning inactivation of the selectivity filter in hKv1.3. We also mutated the nearby P424 to Gly, thinking that this substitution might alter the stability of the turret and impact both inactivation and the effects of NB1.3. Indeed, the P424G mutation close to where NB1.3 binds dramatically enhanced inactivation in control solutions, apparently mimicking the effects of the NB1.3 (Figs. 3d, e, and 4a–c). The P424G mutant also reduced the inhibitory effects of 100 nM NB1.3 (Figs. 3d, e, and 4d), diminishing the extent to which the NB speeds inactivation and shifts the steady-state inactivation relation to more negative voltages (Fig. 4b, c). In WT hKv1.3, slow inactivation is best described by a double exponential fit, and NB1.3 eliminates the slow component and speeds the fast component (Fig. 4a, c)^5^. Interestingly, in the P424G mutant, the *τ* values from a single exponential fit in control solutions is indistinguishable from those of the fast component seen in the WT channel (Fig. 4c) and the *τ* of the fast component observed with the WT channel with NB1.3 bound is only modestly faster in P424G with NB1.3 bound (Fig. 4c), hinting that the P424G mutant and NB1.3 similarly influence the mechanism of inactivation. Mutation of residues Y104 and N33 in NB1.3 that are positioned nearby to P424G in the complex also reduced the inhibitory actions of the NB (Fig. 3f, g), suggesting that interactions of NB1.3 with the turret near P424G are involved in the mechanism by which the NB promotes inactivation.

Motivated by the robust effects of the P424G mutation, we set out to solve the structure of the hKv1.3-P424G mutant using single-particle cryo-EM. We expressed and purified the full-length hKv1.3-P424G channel and solved the structure with an overall resolution of 3.18 Å using C4 symmetry in 150 mM K^+^ (Table S1 and Fig. S6). Evaluation of the density maps shows an overall architecture similar to apo hKv1.3 solved previously^5^, with the S1–S4 domain in an “up” or activated position and the inner S6 gate open. However, in contrast to the D1 and D2 conformations of the selectivity filter seen in apo hKv1.3, only a single dilated conformation similar to D2 was observed in hKv1.3-P424G (Fig. 4e, f). Three densities could be observed within the selectivity filter, two of which could be assigned to K^+^ ions based on their coordination with backbone carbonyl groups in the inner half of the filter (Fig. 4e). The highly conserved hydrogen bonds formed by W436-D449 and T441-Y447 seen in the hKv1.3-H451V mutant are broken, and Y447 rotates behind the filter (Fig. 4f). The cryo-EM density map for the turret near residues 423 and 424 is relatively weak (Fig. S6i), suggesting that the P424G mutation makes the turret more dynamic. In addition, H451 above the selectivity filter exhibits two alternate conformations, one of which is similar to both D1 and D2 conformations of apo hKv1.3, and a second conformation that would clash with Y447 in the conducting conformations (Fig. 4f). Taken together, the results thus far indicate that the interactions of NB1.3 with both the S1–S2 loop and the turret are critical for promoting inactivation of hKv1.3 and suggest that the turret may be particularly important for how NB1.3 regulates inactivation.

### A network of hydrophobic residues between the turret and the selectivity filter

**Fig. 5:**
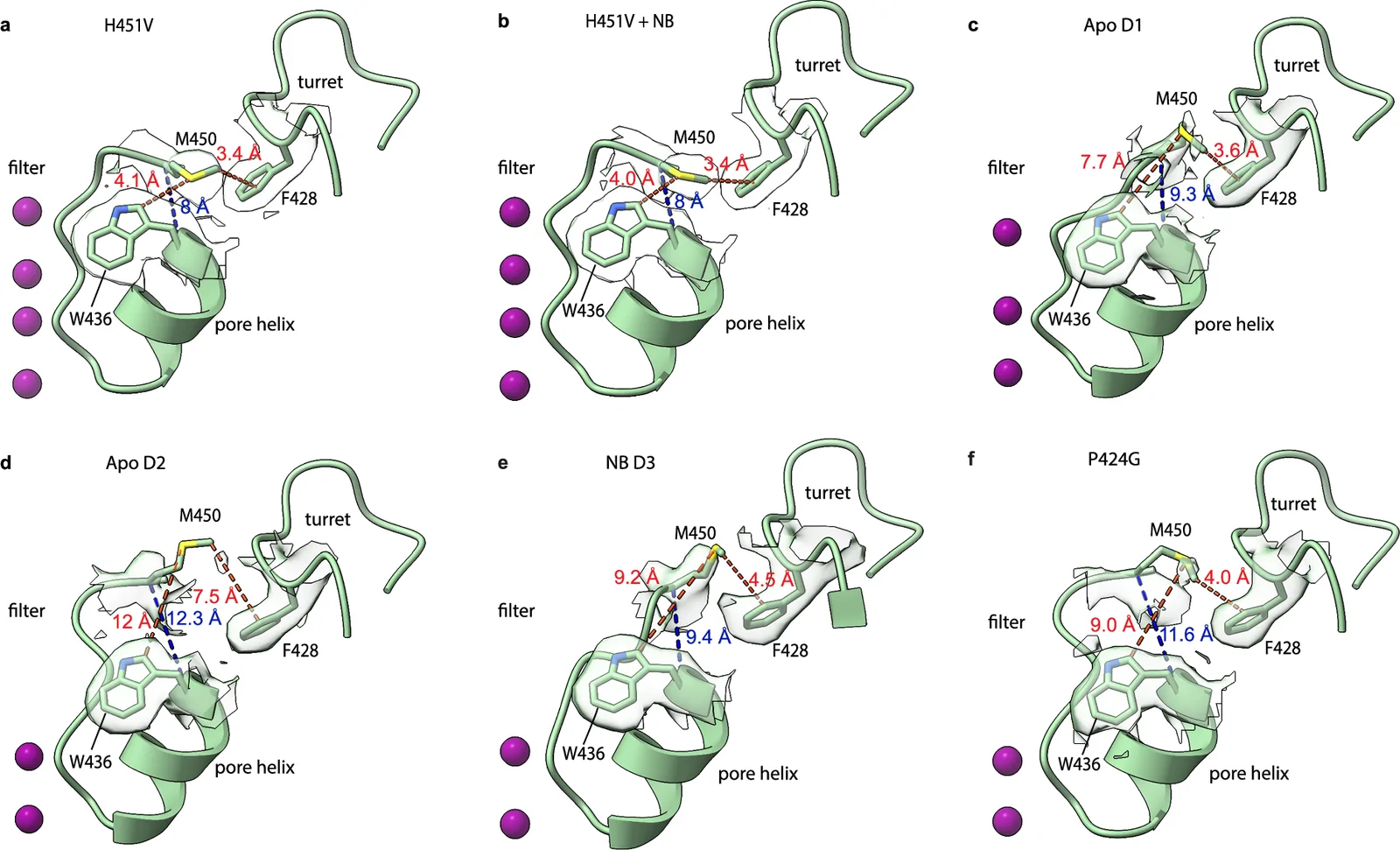
Comparison of hydrophobic side chain interactions between residues in the turret and selectivity filter of hKv1.3 for both conducting and C-type inactivated conformations. **a**, **b** Cryo-EM map and model showing hydrophobic residue interactions between the pore helix and turret of the H451V mutant of hKv1.3 in a conducting conformation without and with NB1.3 bound. **c**, **d** Cryo-EM map and model showing disrupted hydrophobic residue interactions between the pore helix and turret of apo hKv1.3 in D1 and D2 conformations. **e** Cryo-EM map and model showing disrupted hydrophobic residue interactions between the pore helix and turret of the D3 conformation of hKv1.3 with NB1.3 bound. **f** Cryo-EM map and model showing disrupted hydrophobic residue interactions between the pore helix and turret of the P424G mutant of hKv1.3. CA distances between W436 and M450 are colored blue. Distances between SD of M450 and CD1 of W436 and between CE of M450 and CD1 of F428 are colored red.

With several structures of hKv1.3 in conducting conformations (H451V and H451V+NB1.3) and many structures in dilated inactivated states, we looked for clues about how interactions of the NB with the turret might promote inactivation. In addition to the hydrogen bonds involving W436-D449 and T441-Y447 that remain intact only in structures of hKv1.3 in conducting conformations, we identified an additional network of interacting residues between F428 in the turret, M450 in the P-loop and W436 in the pore helix (Fig. 5a, b). Cryo-EM density supporting interactions of these three residues can be clearly seen in the H451V mutant of hKv1.3 in the absence or presence of NB1.3 (Fig. 5a, b). In the D1 conformation of apo hKv1.3 that deviates the least from a conducting conformation, side chain densities for F428 and W436 remain well-defined (Fig. 5c), whereas the backbone density at M450 is displaced and the side chain density is weak. For the D2 conformation of apo hKv1.3, the D3 conformation of NB1.3 bound hKv1.3, and the D2 conformation seen in the P424G mutant of hKv1.3, the backbone density at M450 is displaced, and the density for the side chain is weak, even though the density for the side chains for both F428 and W436 are well-defined. These observations raise the possibility that interactions between F428, M450 and W436 contribute to stabilizing a conducting state and are disrupted during inactivation (Fig. 5d–f). Very subtle reorientations of the phenyl ring of F428 are apparent between these various conducting and inactivated conformations, possibly helping to explain how the interaction of NB1.3 might perturb the network of interacting hydrophobic residues between the turret and ion selectivity filter. All three residues involved in this hydrophobic network are highly conserved among the Kv1 family (Fig. S1), suggesting the network of hydrophobic interactions may be critical for maintaining the conducting conformation of the ion selectivity filter in other Kv1 channels.

**Fig. 6:**
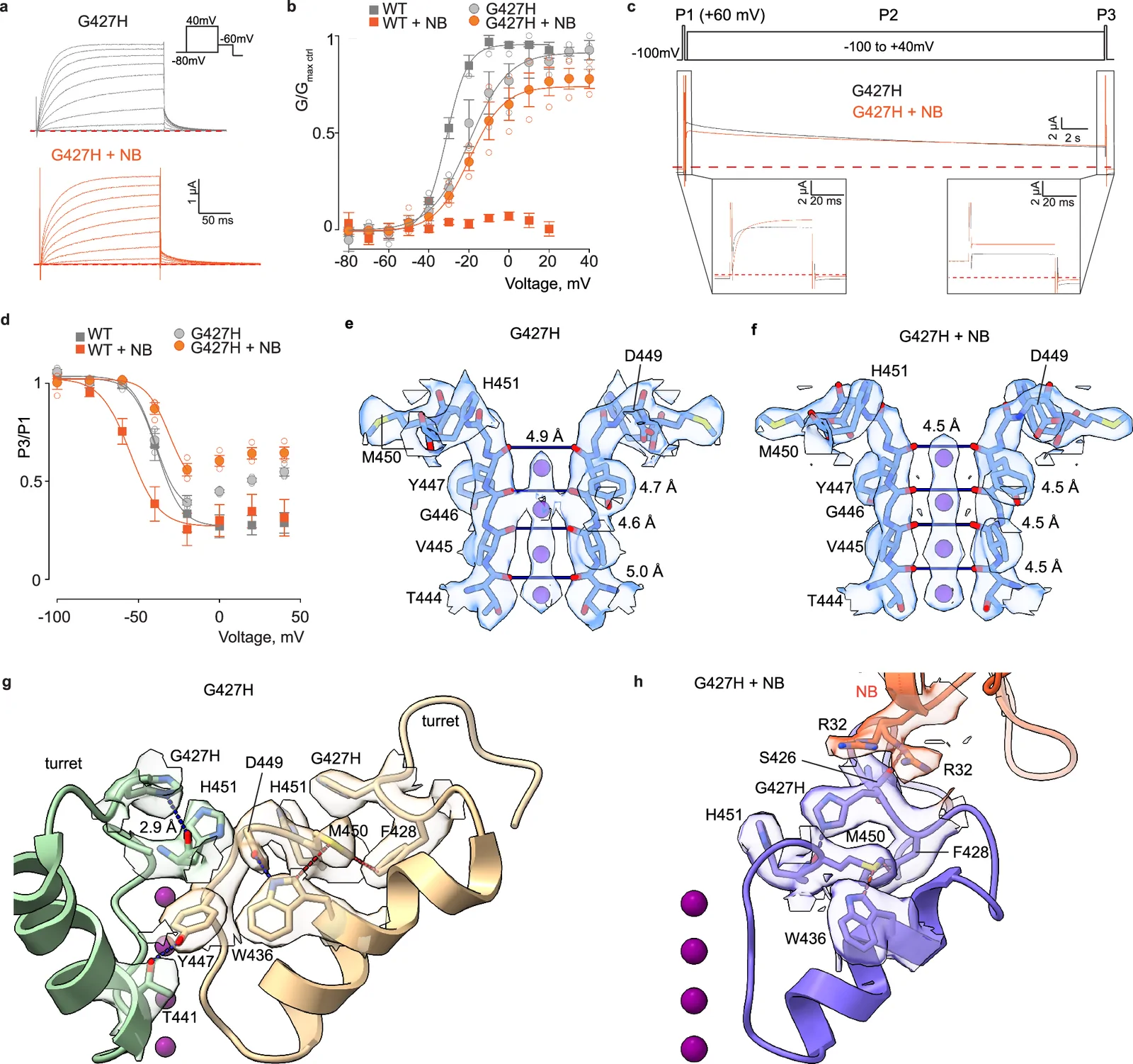
The G427H mutant of hKv1.3 stabilizes a conducting state and interferes with NB1.3, promoting inactivation. **a** Families of currents elicited by depolarization in the absence and presence of 100 nM NB1.3 for the G427H mutant. **b** Plot of normalized conductance (*G*/*G*_max_) obtained from tail current measurements for WT hKv1.3 and the G427 mutant in the absence and presence of 100 nM NB1.3. Smooth curves are Boltzmann fits with *V*_1/2_ and *z* values of −19.8 mV and 2.7 for G427H in the control solution and −19.8 mV and 3 in the presence of NB1.3 (*n* = 3 in 2 independent experiments). Error bars represent S.E.M. WT data are from Fig. 1j. **c** Current traces obtained using a three-pulse protocol without leak subtraction for hKv1.3 G427H in the absence and presence of 100 nM NB1.3. **d** Plot of *P*3/*P*1 for WT hKv1.3 and the G427H mutant in the absence and presence of 100 nM NB1.3. Smooth curves are Boltzmann fits with *V*_1/2_ and *z* values of −39.6 mV and 3.7 for G427H in control solution and −31.2 mV and 3.7 in the presence of NB1.3 (*n* = 3 in 2 independent experiments). Error bars represent S.E.M. WT data are from Fig. 1g. **e** Cryo-EM density and structural model of the ion selectivity filter of the G427H mutant of hKv1.3. **f** Cryo-EM density and structural model of the ion selectivity filter of the G427H mutant of hKv1.3 with NB1.3 bound. **g** Cryo-EM map and model showing hydrophobic residue interactions between the pore helix and turret of the G427H mutant of hKv1.3 in a conducting conformation. Hydrogen bonds are indicated by blue dotted lines and hydrophobic interactions by red dotted lines. **h** Cryo-EM map and model showing hydrophobic residue interactions between the pore helix and turret of the G427H mutant of hKv1.3 in a conducting conformation with NB1.3 bound. Hydrogen bonds are indicated by blue dotted lines and hydrophobic interactions by red dotted lines.

A role of this F428-M450-W436 network between the turret and selectivity filter might help to explain why the P424G mutant dramatically promotes inactivation and interferes with the actions of NB1.3 (Figs. 3d, e, and 4a–c), and possibly why G427H interferes with the actions of NB1.3 (Fig. 3d, e). To better understand the G427H mutant, we further characterized its impact on voltage-dependent activation and inactivation, both alone and in the presence of NB1.3. The G427H mutation exhibited a modest shift of the voltage-activation relationship to more positive voltages, with addition of NB1.3 producing very modest inhibition while slowing both activation and deactivation of the channel (Figs. 6a, b, and S2f), suggesting the NB binds to the channel even though it can no longer promote inactivation. In addition, the G427H mutant exhibited diminished steady-state inactivation in control solutions and remarkably, NB1.3 reduced the extent of inactivation and shifted the steady-state inactivation relation to more positive voltages (Fig. 6c, d), suggesting that in this mutant background the NB interferes with inactivation. As observed with the H451V mutant, G427H also rescued ion conduction in the W436F mutant of hKv1.3 (Fig. S2g), a mutation that in both Shaker and Kv1.3 renders the channels nonconducting by promoting slow inactivation^29,40–44^, suggesting that G427H interferes with inactivation. In addition, the effects of NB1.3 were not altered by increasing the concentration from 100 nM to 1 µM (Fig. S2c, d), suggesting that the lower concentration remains saturating for G427H.

Given the striking impact of the G427H mutation on inactivation and the actions of NB1.3, we decided to solve the structure of the G427H mutant to understand the underlying mechanism. We expressed and purified the hKv1.3-G427H and solved its structure using single particle cryo-EM in 150 mM K^+^ with an overall resolution of 3.54 Å for the entire channel and to 3.05 Å when refinement was focused on the pore domain, in both instances while imposing C4 symmetry (Table S1 and Fig. S7). The overall architecture is similar to apo hKv1.3, with the S1–S4 domains in the “up” or activated position, and the inner S6 gate open. In contrast to apo hKv1.3, however, the ion selectivity filter adopted a conducting conformation similar to that observed in the H451V mutant (Fig. 6e). The oxygen atoms from backbone carbonyl groups in the selectivity filter are positioned to coordinate K^+^ ions and four densities are observed in the filter that can be assigned to K^+^ ions. In addition, the key hydrogen bond networks that stabilize a conducting conformation, including W436-D449 and T441-Y447, remain intact in the G427H mutant (Fig. 6g). The key to understanding how G427H stabilizes a conducting conformation is evident in this structure as the side chain of G427H packs up against M450 to stabilize its interactions with both F428 and W436 and the side chain ND1 in the imidazole side chain of G427H interacts with the backbone carbonyl of H451 above the selectivity filter (Fig. 6g). Thus, the G427H mutation stabilizes the conducting conformation by adding additional interactions that prevent M450 from moving to break its hydrophobic interactions with F428 and W436 (Fig. 6g). Cryo-EM densities for each of these interactions that stabilize a conducting conformation of the selectivity filter are well-defined (Fig. 6g) and support an intact hydrophobic network of interactions between F428, M450, and W436, in addition to those previously characterized between W436 and D449 and T441 and Y447^29,40–44,48^. We also solved the structure of hKv1.3-G427H with NB1.3 bound to an overall resolution of 2.91 Å (Table S1 and Fig. S8). The interface between NB1.3 and the G427H mutant resembled the NB bound hKv1.3 in overall conformation with similar binding interfaces (Fig. S9) but with the selectivity filter in a conducting formation (Fig. 6f) and all of the stabilizing interactions seen in G427H alone remaining intact (Fig. 6g, h). The position of NB1.3 when bound to G427H resembled the outward leaning conformation seen in WT Kv1.3 (Fig. S9) and we saw no evidence of the inward leaning conformation observed in H451V (Fig. 2m, n), further suggesting that these two conformations are unrelated to how the NB enhances inactivation because the outward leaning conformation is seen in WT channels that readily inactivate and in G427H channels that are resistant to inactivation. We think the inward and outward leaning conformations seen for NB1.3 bound to H451V likely represent two snapshots in a partially flexible arrangement. We also observed that the side chain of R32 of NB1.3 displays two alternate conformations, one of which makes a hydrogen bond with the backbone carbonyl group of S426 to prevent movement of H427 and both conformations of R32 would be expected to stabilize H427 through electrostatic repulsion (Fig. 6h). Overall, these results suggest that NB1.3 binds to G427H but instead of promoting inactivation, the NB further stabilizes a conducting state, consistent with the functional effects of the NB on the G427H mutant (Fig. 6a–d, h, and S2f).

**Fig. 7:**
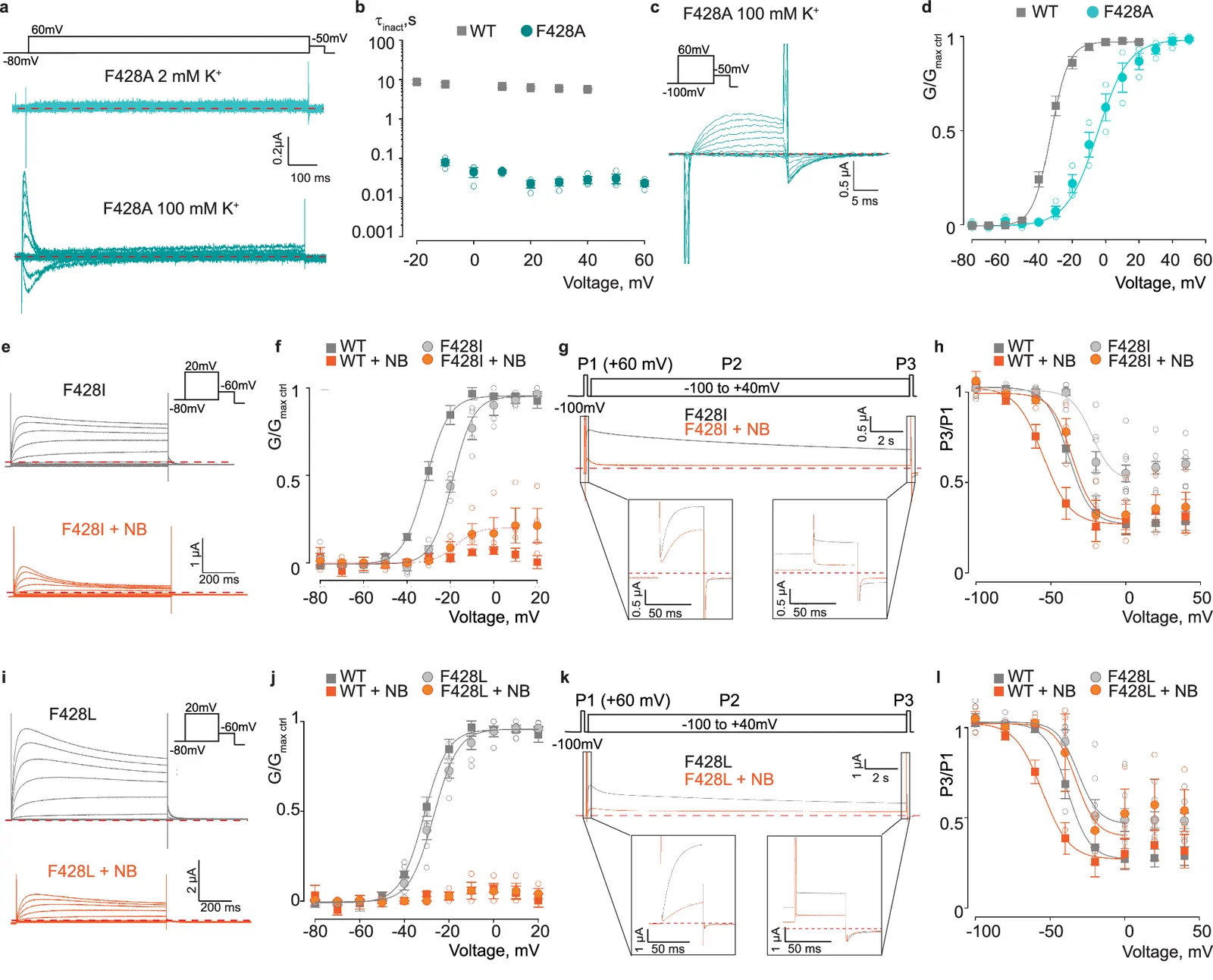
F428A mutants of hKv1.3 promote C-type inactivation. **a** Families of currents elicited by depolarization in the presence of 2 or 100 mM external K^+^ for the F428A mutant of hKv1.3. **b** Inactivation kinetics for WT hKv1.3 and the F428A mutant in 100 mM external K^+^. In both cases, a single exponential function was fit to the data to obtain τ. For F428A, *n* = 3 in 3 independent experiments and for WT *n* = 5 in 3 independent experiments. Error bars represent S.E.M. **c** Family of currents elicited by short depolarization in the presence of 100 mM external K^+^ for the F428A mutant. **d** Plot of normalized conductance (*G*/*G*_max_) obtained from tail current measurements for WT hKv1.3 and the F428A mutant, both with 100 mM external K^+^. Smooth curves are Boltzmann fits with *V*_1/2_ and *z* values of −5 mV and 2.8 for F428A in control solution (*n* = 3 in 3 independent experiments) and −32.9 mV and 4.8 for WT in control solution (*n* = 8 in 4 independent experiments). Error bars represent S.E.M. **e** Families of currents elicited by depolarization without leak subtraction in the absence and presence of 100 nM NB1.3 for the F428I mutant. External K^+^ for (**e**–**l**) was 2 mM. **f** Plot of normalized conductance (*G*/*G*_max_) obtained from tail current measurements for WT hKv1.3 and the F428I mutant in the absence and presence of 100 nM NB1.3. Smooth curves are Boltzmann fits with *V*_1/2_ and z values of −18.9 mV and 5.3 for F428I in control solution and −17.55 mV and 5.1 in the presence of NB1.3 (*n* = 4 in 3 independent experiments). Error bars represent S.E.M. WT data are from Fig. 1j. **g** Current traces obtained using a three-pulse protocol without leak subtraction for hKv1.3 F428I in the absence and presence of 100 nM NB1.3. **h** Plot of *P*3/*P*1 for WT hKv1.3 and the F428I mutant in the absence and presence of 100 nM NB1.3. Smooth curves are Boltzmann fits with *V*_1/2_ and *z* values of −22.5 mV and 3.7 for F428I in control solution (*n* = 8 in 6 independent experiments) and −36 mV and 3.7 in the presence of NB1.3 (*n* = 3 in 3 independent experiments). Error bars represent S.E.M. WT data are from Fig. 1g. **i** Families currents elicited by depolarization without leak subtraction in the absence and presence of 100 nM NB1.3 for the F428L mutant. **j** Plot of normalized conductance (*G*/*G*_max_) obtained from tail current measurements for WT hKv1.3 and the F428L mutant in the absence and presence of 100 nM NB1.3. Smooth curves are Boltzmann fits with *V*_1/2_ and *z* values of −26.8 mV and 4.7 for F428L in control solution (*n* = 6 in 5 independent experiments) and in the presence of NB1.3, currents were too small for reliable fitting (*n* = 3 in 3 independent experiments). Error bars represent S.E.M. WT data are from Fig. 1j. **k** Current traces obtained using a three-pulse protocol without leak subtraction for hKv1.3 F428L mutant in the absence and presence of 100 nM NB1.3. **l** Plot of *P*3/*P*1 for WT hKv1.3 and the F428L mutant in the absence and presence of 100 nM NB1.3. Smooth curves are Boltzmann fits with *V*_1/2_ and *z* values of −31.3 mV and 3.7 for F428L in control solution (*n* = 7 in 6 independent experiments) and −32.8 mV and 3.7 in the presence of NB1.3 (*n* = 3 in 3 independent experiments). Error bars represent S.E.M. WT data are from Fig. 1g.

Of the residues implicated in the hydrophobic network between F428, M450, and W436, only F428 has not been previously implicated in the mechanism of slow inactivation in Kv1 channels. In both Shaker and Kv1.3, mutations equivalent to W436F render these channels nonconducting by promoting slow C-type inactivation, which likely involves breaking of the hydrogen bond between W436 and D449^29,40–44,48^ and disruption of the hydrophobic interactions with M450. In Shaker, mutation of the equivalent of M450 to Cys dramatically speeds slow inactivation^49^, and we observe a similar speeding of inactivation of the M450A mutant in hKv1.3 (Fig. S2h, i), consistent with a key role of M450 in stabilizing a conducting conformation of the selectivity filter. To explore whether F428 also plays a critical role in regulating slow inactivation in hKv1.3, we mutated the residue to Ala and could not observe any evidence that the channel conducted K^+^ ions when studied in low (2 mM) external K^+^ concentrations (Fig. 7a). In contrast, when recording from cells expressing F428A using high external K^+^ ion concentrations (100 mM) to interfere with inactivation, we could measure detectable K^+^ currents that inactivated about two orders of magnitude faster than the WT channel studied in the same external K^+^ concentration (Fig. 7a, b), demonstrating that the F428A mutant dramatically alters inactivation. The mutant also exhibited a voltage-activation relationship that was shifted to more positive voltages (Fig. 7c, d), suggesting that this region of the turret may also influence activation. We also studied F428I and F428L mutations and found that both could conduct K^+^ even with low external K^+^ concentrations (Fig. 7e, i), both mutants inactivate, albeit less completely than WT hKv1.3 (Fig. 7g, h, k, l), and NB1.3 can promote inactivation, but in subtle ways that are quantitatively different than the WT channel (Fig. 7e–l). Taken together, these results support a critical role of F428 and the hydrophobic network involving M450 and W436 in regulating inactivation of hKv1.3 and in mediating the actions of NB1.3.

## Discussion

The goal of the present study was to understand how the binding of NB1.3 to the external surface of hKv1.3 inhibits the channel by promoting inactivation. Mutagenesis of both the S1–S2 loop with the S1–S4 voltage-sensing domain and of the turret within the pore domain demonstrate that interactions of NB1.3 with both regions are required to promote inactivation (Fig. 3). Mutations at the interface between the S1–S2 loop and NB1.3 diminish the extent to which the NB promotes inactivation (Fig. 3). Importantly, the Y265G mutant disrupted speeding of inactivation by the NB without altering the inactivation properties of hKv1.3 in the absence of the NB (Fig. S5c, d). We therefore conclude that interactions of NB1.3 with the S1–S2 loop are required to promote inactivation but that the S1–S2 loop does not appear to have a pronounced influence on inactivation. In contrast, mutations in the turret region can either promote or interfere with inactivation in the absence of the NB and diminish the extent to which NB1.3 promotes inactivation (Figs. 3, 4, and 6), more directly implicating the turret in the mechanism by which NB1.3 promotes inactivation. The key to understanding how the interaction of NB1.3 with the turret promotes inactivation of the ion selectivity filter came from comparing structures of H451V mutations of hKv1.3 in conducting conformations (Fig. 2) with structures of hKv1.3 in inactivated states in the absence or presence of NB1.3^5^ or with mutations that promote inactivation (Figs. 4 and S10, S11). That comparison revealed the presence of a network of interacting hydrophobic residues involving F428 in the turret, M450 in the P-loop and W436 in the reentrant pore helix that were maintained when the ion selectivity filter resides in a conducting state (Figs. 5 and S10, S11). The importance of this network in the actions of NB1.3 are further supported by the structure of the G427H mutation in a conducting conformation where the hydrophobic network is intact (Figs. 6, and S10, S11), by functional experiments showing that NB1.3 does not promote inactivation in that mutant (Fig. 6) and by mutations at F428 and M450 dramatically speeding inactivation in the absence of the NB (Figs. 7 and S2h, i).

**Fig. 8:**
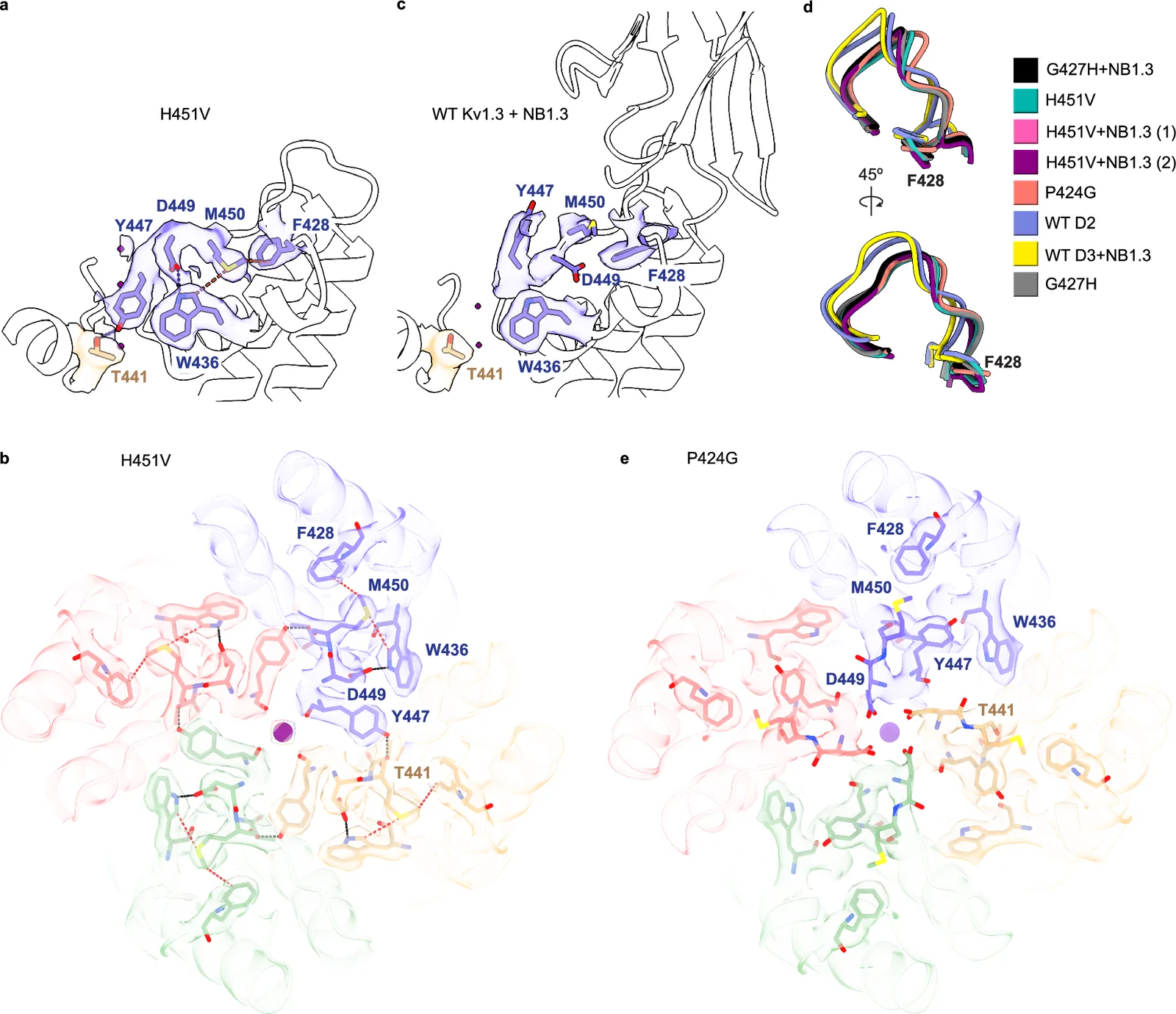
Structural changes in the interaction of hydrophobic residues between the turret and selectivity filter during C-type inactivation. Cryo-EM density and model of the H451V mutant of hKv1.3 view from the side (**a**) or from the external side of the membrane (**b**). Key residues are labeled, hydrogen bonds are indicated by blue dotted lines and hydrophobic interactions by red dotted lines. **c** Side view of cryo-EM density and model of hKv1.3 with NB1.3 bound with key residues indicated. Note the absence of key hydrogen bonds and hydrophobic interactions illustrated in (**a**). **d** Superimposing models for the turret from this study and our previous study^5^. **e** Cryo-EM density and model of the P242G mutant of hKv1.3 viewed from the extracellular side of the membrane with key residues indicated. Note the absence of key hydrogen bonds and hydrophobic interactions illustrated in (**c**).

The present results extend our understanding of the key network of interactions within the external pore of hKv1.3 channels that help to stabilize a conducting state and that rearrange during slow C-type inactivation (Fig. 8). Two previously recognized interactions that stabilize the ion selectivity filters of Kv channels in a conducting state are the intersubunit interaction of Y447 with T441 and the intrasubunit interaction of D449 with W436 (Fig. 8a, b)^40^. These interactions can be seen to break as either Shaker or hKv1.3 channels inactivate (Fig. 8c, e)^5,29,35^, and mutations of these positions exhibit faster C-type inactivation, in some cases so strongly that the channels do not conduct ions (e.g., W434F in Shaker or W436F in Kv1.3)^29,40–44,48^. W436 plays a particular critical role in stabilizing a conducting state because it hydrogen bonds with D449 and interacts with M450 in the hydrophobic network terminating in the turret at F428 (Fig. 8a, d). Our structures suggest that binding of NB1.3 to the turret likely produces relatively subtle changes in the structural dynamics of the turret to weaken the interaction of F428 with M450. Superimposing the available structures of hKv1.3 reveals that during inactivation, the turret adopts a distinct conformation that produces a subtle deviation in the orientation of F428 to weaken the hydrophobic network with M450 and W436 (Fig. 8d). The conformations of the turret in the conducting conformations seen in H451V and G427H in the absence or presence of NB1.3 all superimpose well and exhibit similar orientations of F428 (Fig. 8d). The turret and F428 in the rapidly inactivating P424G mutant differ from the other conducting and inactivated structures (Fig. 8d), likely reflecting the radical nature of that mutation and the expectation that it would introduce flexibility into the backbone of the turret. Inspection of cryo-EM maps reveal that the density for the turret region is the best defined in the H451V and G427H mutants (Figs. S3, S4, S7, S8, and S11), and much more poorly defined in P424G (Fig. S6) and the previously published structures of hKv1.3 channels in inactivated states^5^, suggesting that a dynamic turret favors inactivation and a more stable turret favors a conducting state, inferences that would be interesting to explore in future studies. It is also notable that cryo-EM density for the side chain of M450 is resolved in all structures of hKv1.3 with the filter in a conducting state (Figs. 2d, k, 5a, b, and 6e–h) and unresolved in structures of hKv1.3 in an inactivated state (Figs. 2a–c, 4e, f, and 5c–f).

It is interesting that the network identified here involving F428, M450, and W436 is fully conserved in all human Kv1 channels (Fig. S1), raising the possibility that this network stabilizes the conducting state in Kv1 channels and tunes both the inactivation and the pharmacological properties of different Kv1 channels. The G427H mutation that was critical in elucidating how NB1.3 promotes inactivation in hKv1.3 is also notable because five of the eight Kv1 channels contain a His at the equivalent to G427, including Kv1.1, Kv1.4, Kv1.5, Kv1.7, and Kv1.8 (Fig. S1). Although the sensitivity of Kv1 channels to NB1.3 is not available for all subtypes, both Kv1.4 and Kv1.5 are insensitive to NB1.3^6^. Kv1.2 is also insensitive to NB1.3^6^, but in this case the insensitivity can be explained because this subtype contains a Val at the position corresponding to H451 (Fig. S1), a substitution that stabilizes the conducting state and disrupts the effects of NB1.3 in promoting inactivation (Fig. 2). Many small molecule inhibitors have been developed that selectively target hKv1.3 and achieve selectivity at least in part by interacting with the mechanism of slow inactivation. For example, PAP-1 is known to inhibit hKv1.3 by interacting with the mechanism of inactivation and the apparent affinity of PAP-1 for hKv1.3 is one to two orders of magnitude higher compared to Kv1.1 to 1.7^50^. Four of these PAP-1 insensitive subtypes contain a His at the equivalent of G427 (Kv1.1, Kv1.4, Kv1.5, and Kv1.7), and one (Kv1.2) contains a Val at the position corresponding to H451 (Fig. S1). A similar pattern of subtype selectivity has also been reported for Psora-4^51^. It will be interesting to explore whether stabilization of the F428-M450-W436 network by His at positions equivalent to G427 plays a critical role in determining the properties of slow C-type inactivation in different Kv1 channels and the extent to which these influence their pharmacological sensitivities.

Our collective findings on the interaction between NB1.3 and hKv1.3 provides motivation for generating function-altering NBs that target other tetrameric cation channels that have a similar architecture to Kv1.3^52^, including not only K^+^ selective channels, but also voltage-activated Na^+^ and Ca^2+^ channels, cyclic nucleotide-gated channels and transient receptor potential (TRP) channels. Most of the channels in this larger family contain S1–S4 domains that sense voltage or other chemical activators, and they are domain-swapped with respect to the pore domain, enabling a nanobody to bind to the extracellular surface at the interface between subunits to interact simultaneously with the S1–S4 domain and the turret within the pore domain. Both regions in tetrameric cation channels would be ideal sites for modulating the functional properties of these channels. While our focus on understanding how NB1.3 promotes inactivation of hKv1.3 led to the discovery of a key role of the F428-M450-W436 network in controlling inactivation, we also uncovered additional functional effects of NB1.3, suggesting that it can modulate other aspects of Kv channel function. For example, NB1.3 retained some inhibitory activity in both the H451V and G427H mutants that disrupted slow inactivation (Figs. 2i, j, and 6a, b), and the NB slowed closing for both channels (Figs. 2h, i, and S2f), suggesting that the NB can influence the closed to open transition and possibly the single channel conductance from its perch on the external surface of hKv1.3. It will be exciting to see how the development of new NBs targeting this larger family of tetrameric ion channels^1,3,6^ might lead to powerful new mechanistic tools and therapeutics.

## Methods

### Ethical statement

The animal care and experimental procedures were performed in accordance with the Guide for the Care and Use of Laboratory Animals and were approved by the Animal Care and Use Committee of the National Institute of Neurological Disorders and Stroke (animal protocol number 1253).

### Animals

Female *Xenopus laevis* frogs, 1–2 years of age.

### Site directed mutagenesis, expression, and purification of human Kv1.3 mutants

The previously designed human Kv1.3 protein expression gene construct in the pGEM vector was utilized for the generation of various mutations performed by Genescript services. Mutants P424G, G427H and H451V were subsequently cloned into the pEZT-BM BacMam expression vector^53^, and at the C-terminus was fused to a 3C protease recognition site followed by the mVenus fluorescent protein and a Twin-Strep affinity tag. Protein expression for P424G and H451V were carried out by using Expi293™ Expression System Kit (Gibco) following manufacturer’s instructions. Briefly, Expi293F™ cells were grown (1.6 L or 3.2 L) at 37 °C and 8% CO_2_ with shaking to a density of 3.0 ×  10^6^ cells/mL in Expi293™ Expression Medium (Gibco) and antibiotic Penicillin-Streptomycin (Gibco). Transfection of plasmid DNA was done through ExpiFectamine™ 293 reagent. After 18–22 h post- transfection, ExpiFectamine™ 293 Transfection Enhancer 1 and 2 were added, and flasks were returned to the 37 °C incubator and 8% CO_2_ with shaking. Cells were collected 72 h after transfection by low-speed centrifugation of 6200 × *g* for 20 min at 4 °C, flash-frozen in liquid nitrogen, and stored at −80 °C. Baculovirus-mediated mammalian expression^54^ was used for G427H mutant protein production. Briefly, the G427H construct was transformed into DH10Bac cells to produce bacmid, which was then transfected into Sf9 cells grown in ESF 921 media (Expression Systems). P1 and P2 virus production was monitored using GFP fluorescence from the pEZT-BM vector until virus harvesting. tsA201 cells at approximately 1.5 million cells per ml in freestyle medium with 2% FBS were transduced with 10% (v/v) of the P2 virus and incubated at 37 °C CO_2_ incubator. To boost the protein expression, 10 mM sodium butyrate was added at approximately 16 h post-transduction. The culture was continued at 30 °C in a CO_2_ incubator for another 24 h, and the cells were harvested by centrifugation and frozen at −80 °C until use.

The cell pellets were first resuspended in ice-cold resuspension buffer, which consisted of 50 mM Tris (pH 7.5), 150 mM KCl, 2 mM DTT, a protease inhibitor tablet (Sigma), 1 mM PMSF, 0.5 mM EDTA, and 25 μg/ml DNAse. The mixture was manually pipetted until no clumps remained. Subsequently, the cells were disrupted on ice using a QSonica Q700 sonicator in three rounds of 60 s each, at a power level of 40 (Pulse ON: 15 s, pulse OFF: 45 s). The lysate was then clarified by subjecting it to low-speed centrifugation at 7200 × *g* for 20 min. The membranes were then isolated by ultracentrifugation at 125,000 × *g* for 2 h. The isolated membranes were resuspended and homogenized in a buffer comprising 20 mM Tris (pH 7.5), 150 mM KCl, a protease inhibitor tablet (Sigma), 1 mM PMSF, and 0.5 mM EDTA. For protein extraction, 50 mM n-dodecyl-β-D-maltopyranoside (DDM) with 4 mM cholesteryl hemisuccinate (CHS) were added to the homogenized membranes. This mixture was nutated for 1 h at 4 °C, and then ultracentrifugation was done at 125,000 × *g* for 50 min to remove any insoluble material. The resulting supernatant was filtered through a 0.45 μm filter and then bound to a 5 ml prepacked Strep-Trap HP column (Cytiva) that was previously equilibrated with running buffer containing 20 mM Tris (pH 7.5), 150 mM KCl, 0.5 mM EDTA, and 2 mM DDM with 0.16 mM CHS. To remove any bound heat shock proteins, the bound protein was washed with running buffer containing 10 mM MgCl_2_ and 5 mM ATP. The elution step was carried out using a running buffer consisting of 10 mM desthiobiotin (IBA). Elution fractions were collected, and their protein content was analyzed by SDS-PAGE. Subsequently, the elution fractions containing the protein of interest were concentrated and loaded onto a Superose 6 Increase 10/300 GL (Cytiva) column. The column was equilibrated with a buffer comprising 20 mM Tris (pH 7.5), 150 mM KCl, 2 mM DTT, 0.5 mM EDTA, and 0.5 mM DDM with 0.04 mM CHS. Elution fractions were collected, analyzed by SDS-PAGE, and peak fractions were concentrated and used for cryo-EM experiments.

### Nanobody mutations, expression, and purification

Eight alanine mutations of the NB1.3 (A0194009G09) nanobody were generated (R32, N33, R51, K76, W99, F103, Y104, and E105) using the QuikChange Lightning Multi Site-Directed Mutagenesis Kit. Expression and purification of NB1.3 was previously described^5^ and the same procedure was applied for all the alanine mutants. Briefly, BL21 DE3 cells were transformed with the plasmid and grown at 37 °C in TB media supplemented with 1 mM MgCl_2_, 0.01 % glucose, and kanamycin (25 μg/ml) and induced by 1 mM IPTG when the cells reached OD_600_ of 0.7. After 3 h, the cells were harvested and pellets stored at −80 °C. To purify the protein, cell pellets were resuspended in the buffer containing 20 mM Tris (pH 7.5), 150 mM KCl, 1 mM PMSF, 5 mM MgCl_2_, 0.05 mg/mL DNAse and 0.2 mg/mL lysozyme and then sonicated on ice. Lysate was clarified by centrifugation, and affinity purification was done using TALON cobalt resin (Takara). Clarified lysate was loaded on the column, washed with 10 column volumes of running buffer 20 mM Tris (pH 7.5), 150 mM KCl and 5 mM imidazole, then protein was eluted with running buffer supplemented with 50 mM imidazole. The wild type nanobody and alanine mutants were further purified using gel filtration with buffer containing 20 mM Tris (pH 7.5), 150 mM KCl, 0.5 mM EDTA. Peak fractions were then pooled and concentrated.

### Cryo-EM sample preparation and data acquisition

Samples were prepared using G427H mutant protein (5 mg/ml) without a binding partner or with nanobody at 2.2 mg/ml (3:1 molar ratio of nanobody to Kv subunit), H451V mutant protein (4.3 mg/mL) without a binding partner, or with nanobody at 1.88 mg/mL (3:1 molar ratio of nanobody to Kv subunit) and 2.1 mg/ml for P424G mutant proteins. UltraAuFoil 1.2/1.3 300 mesh grids (Quantifoil) were plasma treated, and vitrified samples were prepared by adding a 3 μL droplet of sample solution to a grid, then blotting (2 s blot time, 0 blot force) and plunge-freezing in liquid ethane using a Vitrobot Mk IV (Thermo Fisher).

Single particle images were collected with a Titan Krios electron microscope (Thermo Fisher) operated at 300 kV and a nominal magnification of ×105,000 and equipped with a K3 camera (Gatan) set in super-resolution mode (0.4125 Å or 0.415 Å pixel size). Leginon^55,56^ was used for automated collection of images with ice thickness ranging between 20 and 120 nm (H451V,H451V+NB1.3,P424G) and serial EM^57^ was used for G427H and G427H+NB1.3. Movies were collected at nominal defocus values of ~0.2–2.4 μm and dose-fractionation into 40 frames (G427H and G427H+NB1.3), 50 frames (H451V) and 60 frames (H451V+NB1.3 and P424G) with a total exposure time of 2.0 s (G427H, G427H + NB1.3, H451V) and 1.8 s for (H451V+NB1.3 and P424G). Total dose was 50 (G427H), 51.6 (G427H+NB1.3), 58.58 (H451V), 52.16 (H451V+NB1.3) and 51.80 (P424G) electrons per Å^2^, respectively.

### Image processing and structure analysis

Relion version 3^58^ and Cryosparc version 4^59^ were used in all steps of data processing. Movie stacks were corrected for beam induced motion with two-fold binning and dose-weighted and the resulting images were used for contrast transfer function (CTF) estimation with CTFFIND4 in Relion^60^. Datasets were then processed as follows. For all the datasets, an initial particle set was picked with the reference-free Laplacian-of-Gaussian (LoG) tool in Relion, and particles were extracted with a box size of 320 pixels (H451V, P424G, and G427H) and 416 (H451V+NB1.3, G427H+NB1.3), then downscaled to 128 pixels. These particles were imported into cryoSPARC and processed with 2D classification, ab initio reconstruction and heterogeneous refinement to generate a well-defined particle subset. The subset was re-extracted in Relion without binning, and the 3D model generation tool in Relion was used to create a template map for particle picking. Particle picking was done with an interparticle distance of 150 Å to avoid picking duplicates and yielded 558,554, 1,061,839, 2,830,248, 1,734,978, and 1,517,810 particles for H451V, H451V+NB1.3, P424G, G427H, and G427H+NB1.3, respectively. The new particle sets were extracted with a box size of 320 (H451V, P424G) and 416 (H451V+NB1.3), imported into cryoSPARC. Initial 3D models were generated using ab initio reconstruction then followed by multiple rounds of heterogeneous refinement to isolate a subset of particles, which were refined with homogeneous and nonuniform refinement with C1 and C4 symmetry. These particles were taken to Relion for Bayesian polishing and back to cryoSPARC for final nonuniform refinement with C4 symmetry for map generation. The H451V+NB1.3 dataset was further processed by ab initio reconstruction and heterogeneous refinement to isolate maps for the inward and outward nanobody conformations 1 and 2. For G427H to improve TMD map resolution local refinement with a mask on pore region was performed in cryoSPARC.

### Structural modelling and refinement

A homology model for P424G, G427H, and H451V was generated using PDB 7SSX (human Kv1.3 wild type structure) and 8DFL (human Kv1.3 and nanobody complex structure) for G427H+NB1.3 and H451V+NB1.3. The homology models were docked into each of maps using UCSF Chimera^61^, and the model for each structure was refined using PHENIX^62^ with geometric and rotamer restraints and then manually corrected in COOT^63^ to fit the density. The final model was evaluated in PHENIX and wwPDB validation system. The extracellular VSD linkers were not resolved in the maps so were not modeled in G427H, H451V and P424G, but for G427H+NB1.3 and H451V+NB1.3, the first segment of the S1–S2 loop, which was stabilized by nanobody binding complex could be modeled. Structural figures were generated using UCSF ChimeraX^64^.

### Electrophysiological recordings

The cDNA encoding the full-length hKv1.3 and hKv1.3-Δ52^44^ (used in Fig. S2e, g) was cloned into a pGEM vector^65^, linearized with NheI and transcribed by using a mMESSAGE mMACHINE^™^ T7 Transcription Kit (Invitrogen/ThermoFisher). Female Xenopus laevis animals were housed, and surgery was performed according to the guidelines of the National Institute of Neurological Disorders and Stroke, Animal Care and Use Committee (ACUC) protocol number 1253. Oocytes were removed surgically and incubated with agitation for 1 h in a solution containing (in mM) 82.5 NaCl, 2.5 KCl, 1 MgCl_2_, 5 HEPES, pH 7.6 (with NaOH), and collagenase (1.2 mg/ml; Worthington Biochemical, Lakewood, NJ). Defolliculated oocytes were injected with 50 nl of channel RNA (~500 ng/ml) and maintained at 16 °C for 1–3 days in an ND96 oocyte maintenance buffer, containing (in mM): 96 NaCl, 2 KCl, 5 HEPES, 1 MgCl_2_ and 1.8 CaCl_2_ plus 50 mg/ml gentamycin, pH 7.6 with NaOH. Voltage-clamp recordings were performed using the two-electrode voltage-clamp recording techniques (OC-725C; Warner Instruments) with a 150 µl recording chamber that was perfused continuously. Data were filtered at 1 kHz and digitized at 10 kHz using a Digidata 1550B AD converter and pCLAMP 10 software (Axon; Molecular Devices). Microelectrode resistances were 0.2–0.8 MΩ when filled with 3 M KCl. For recording macroscopic Kv channel currents with low external K^+^ for the majority of experiments presented here, the external recording solution contained (in mM): 2 KCl, 98 NaCl, 5 HEPES, 1 MgCl_2_, and 0.3 CaCl_2_, pH 7.6, with NaOH. For recording macroscopic Kv channel currents with high external K^+^ (Figs. 7a–d and S2e, g, h), the external recording solution contained (in mM): 100 KCl, 5 HEPES, 1 MgCl_2_, and 0.3 CaCl_2_, pH 7.6, with NaOH. The nanobody and nanobody mutants were added to the control recording solution at the indicated concentrations using continuous perfusion of the recording chamber. All experiments were performed at room temperature (22 °C). Leak and background conductances were subtracted for tail current measurements by arithmetically deducting the end of the tail pulse of each analyzed trace. In most instances, Kv channel currents shown have been leak subtracted using a P/−4 subtraction protocol^66^. We indicated in the figure legends instances where leak subtraction was not used (typically longer inactivation protocols).

*G*–*V* relationships were obtained by measuring tail currents, and a single Boltzmann function was fit to the data according to:

1 $$\frac{I}{{I}_{\max }}=\left(1+{e}^{-{zF}(V-{V}_{\frac{1}{2}})/{RT}}\right)$$

where *z* is the equivalent charge, *V*_1/2_ is the half-activation voltage, *F* is Faraday’s constant, *R* is the gas constant and *T* is temperature in Kelvin. Steady-state inactivation relationships obtained using three-pulse protocols (e.g., Fig. 1e) and plotting *P*3/*P*1 vs. *V* (e.g., Fig. 1g) were also fit with the Boltzmann equation. For the WT channel and most mutants, an interpulse interval of 60 s was sufficient for full recovery from inactivation between consecutive pulses, resulting in *P*3/*P*1 values near unity at negative *P*2 voltages. For P424G (Fig. 4b), even with an interpulse interval of up to 80 s, recovery from inactivation was incomplete, and therefore, *P*3/*P*1 values at negative *P*2 voltages were ~0.8.

For all data obtained in this study, time constants (*τ*) for inactivation were obtained by fitting a single exponential function to the traces for depolarization between +20 and +50 mV. The traces were fitted from the beginning of the decaying phase to the end of the depolarizing trace using the following equation:

2 $$f\left(t\right)={\sum }_{i=0}^{n}{A}_{i}{e}^{-t/{\tau }_{i}}+C$$

In Fig. 4c, data are shown from our earlier study^5^ where a double-exponential fit was used to fit inactivation of WT hKv1.3 for the purpose of illustrating that the fast time constant in WT in the control solution is identical to the time constant obtained from a single exponential fit for P424G in control conditions.

### Reporting summary

Further information on research design is available in the Nature Portfolio Reporting Summary linked to this article.

## Supplementary information


Supplementary Information
Description of Additional Supplementary Files
Supplementary Movie 1
Reporting Summary
Transparent Peer Review File


## Source data


Source Data


## Data Availability

All data needed to evaluate the conclusions in the paper are present in the paper and/or the Supplementary Materials. Models of hKv1.3 mutant proteins have been deposited in the Protein Data Bank with accession codes 9CLA for hKv1.3 H451V mutant, 9CON for hKv1.3 H451V mutant in complex with NB1.3 conformation1, 9CTY for hKv1.3 H451V mutant in complex with NB1.3 conformation2, 9CLV for hKv1.3 P424G mutant, 9EEF for hKv1.3 G427H mutant and 9EI0 for hKv1.3 G427 mutant in complex with NB1.3. Maps of hKv1.3 mutants have been deposited in the Electron Microscopy Data Bank (EMDB) under accession codes EMD-45668 (hKv1.3 H451V mutant); EMD-45786 (hKv1.3 H451V mutant in complex with NB1.3 conformation1); EMD-45922 (hKv1.3 H451V mutant in complex with NB1.3 conformation2); EMD-45731 (hKv1.3 P424G mutant); EMD-47955 (hKv1.3 G427H mutant); EMD-47962 (hKv1.3 G427H pore domain); EMD-48072 (hKv1.3 G427H in complex with NB1.3). Additional datasets used in this study include Protein Data Bank accession code 7SSV, 7SSX, 7SSY, 7SSZ, and 8DFL. The source data underlying the electrophysiology data in Figs. 2–4, 6, 7, and S2 and S5 are provided as a Source data file. A019400G09 nanobody was recombinantly expressed in *E.coli* Bl-21DE3. Nanobody Sequence was obtained from the patent [https://patents.google.com/patent/CA2951443A1/en]. Source data are provided with this paper.
