# Supplementary material for: Nanobody regulation of C-type inactivation in Kv1.3 channels
Source: Nat Commun. 2026 Aug 4;17:9379. doi: 10.1038/s41467-026-74630-8 (PMC13534573; doi:10.1038/s41467-026-74630-8)
Supplement: Supplementary file 1 [file 41467_2026_74630_MOESM1_ESM.pdf]

Supplementary Information

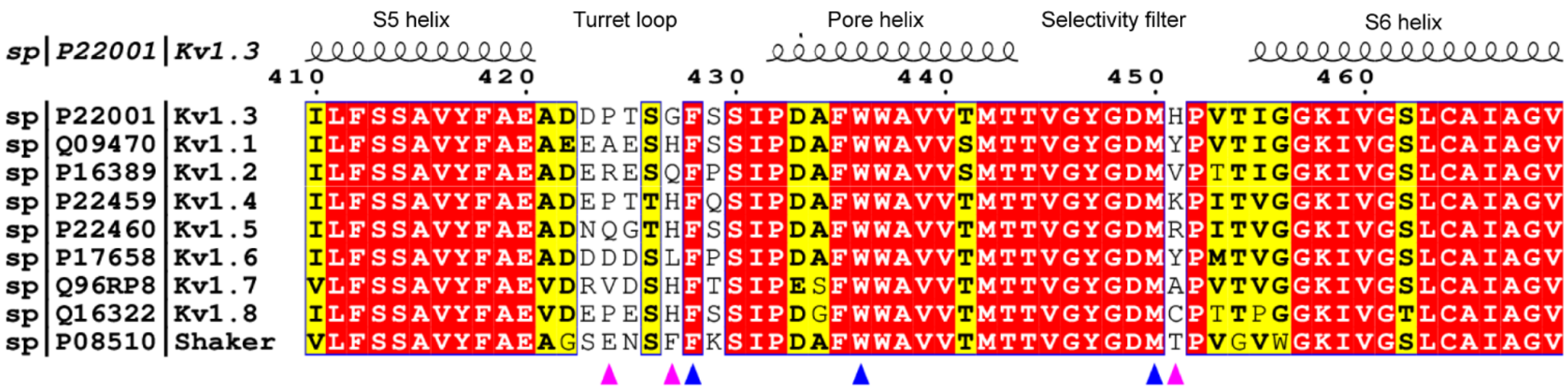

Fig. S1| Sequence alignment for Kv1 channels and the Shaker Kv channel.

Sequence alignment generated by clustal omega of all Kv1 members and the Shaker Kv channel corresponding to the region corresponding to residues 410-469 of hKv1.3. Secondary structural features are indicated above the sequence are from the apo structure of hKv1.3 and detected using ESPript. Pink triangles indicate the positions where mutant structures were solved. Blue triangles indicate the positions of conserved residues involved in the hydrophobic network identified in this study.

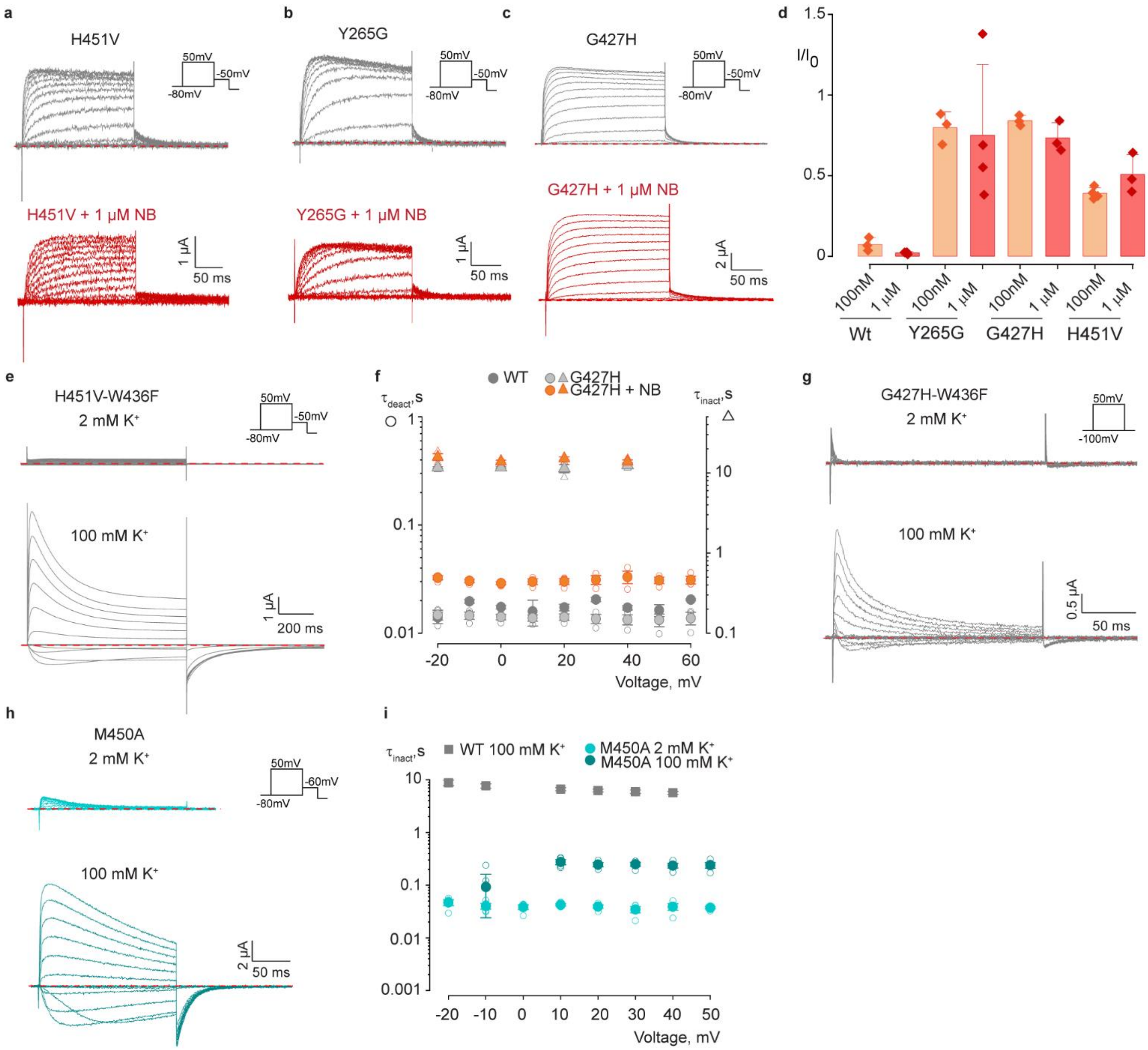
a
H451V
50mV
-50mV
-80mV
H451V + 1 μM NB
1 μA
50 ms
b
Y265G
50mV
-50mV
-80mV
Y265G + 1 μM NB
1 μA
50 ms
c
G427H
50mV
-50mV
-80mV
G427H + 1 μM NB
2 μA
50 ms
d
I/I0
1.5
1
0.5
0
100nM
1 μM
Wt
Y265G
G427H
H451V
e
H451V-W436F
2 mM K+
50mV
-50mV
-80mV
100 mM K+
1 μA
200 ms
f
WT
G427H
G427H + NB
τdeact, s
τinact, s
1
0.1
0.01
10
1
0.1
-20
0
20
40
60
Voltage, mV
g
G427H-W436F
2 mM K+
50mV
-100mV
100 mM K+
0.5 μA
50 ms
h
M450A
2 mM K+
50mV
-60mV
-80mV
100 mM K+
2 μA
50 ms
i
τinact, s
WT 100 mM K+
M450A 2 mM K+
M450A 100 mM K+
10
1
0.1
0.01
0.001
-20
-10
0
10
20
30
40
50
Voltage, mV

← **Fig. S2**

a) Families of currents elicited by depolarization in the absence and presence of 1 µM NB1.3 for the hKv1.3 H451V mutant. **b)** Families of currents elicited by depolarization in the absence and presence of 1 µM NB1.3 for the hKv1.3 Y265G mutant. **c)** Families of currents elicited by depolarization in the absence and presence of 1 µM NB1.3 for the hKv1.3 G427H mutant. **d)** Plot of normalized currents at 0 mV (I/I_0) for WT hKv1.3 and different hKv1.3 mutants at 100 nM and 1 µM NB1.3 obtained from data like those in panels a-c and Fig. 2d,e. Values for n and independent experiments (n/i) were: for Kv1.3 WT at 100 nM (3/3) and 1 µM (4/3), for Y265G at 100 nM (4/3) and 1 µM (4/4), for G427H at 100 nM (3/3) and 1 µM (3/2), for H451V at 100 nM (4/4) and 1 µM (5/4). Error bars represent S.E.M. **e)** Families of currents elicited by depolarization in the presence of 2 mM and 100 mM external K^+ for the hKv1.3-D52 H451V-W436F double mutant. **f**) Plot of time constants (t) for inactivation (triangles) or deactivation (circles) for G427H in the absence or presence of 100 nM NB1.3. A single exponential function was fit to inactivation of G427H using current traces elicited during P2. For G427H, n = 3 in 2 independent experiments and for WT data are from Fig. 1h. A single exponential function was fit to the tail currents like those in Fig. 5a for deactivation of G427H (n=3 in 3 independent experiments). Error bars represent S.E.M. and WT data are from Fig. 1h. **g)** Families of currents elicited by depolarization in the presence of 2 mM and 100 mM external K^+ for the hKv1.3-D52 G427H-W436F double mutant. **h**) Families of currents elicited by depolarization in the hKv1.3 M450A mutant in 2 mM and 100 mM external K^+. **i)** Inactivation kinetics of WT hKv1.3 and the M450A mutant in the presence of 100 mM external K^+ for WT and both 2 mM and 100 mM K^+ for M450A. In all cases a single exponential function was fit to the data to obtain t. For M450A, n = 4 in 2 independent experiments. Error bars represent S.E.M. and for WT data are from Fig. 6b.

Table S1 | Cryo-EM data collection, refinement and validation statistics for structures of the hKv1.3 channel.

	Kv1.3 H451V<br><br>EMDB ID:45668<br>PDB ID: 9CLA	Kv1.3 H451V with NB1.3 conformation1<br>EMDB ID:45786<br>PDB ID: 9CON	Kv1.3 H451V with NB1.3 conformation2<br>EMDB ID:45922<br>PDB ID: 9CTY	Kv1.3 P424G<br><br>EMDB ID:45731<br>PDB ID: 9CLV	Kv1.3 G427H<br><br>EMDB ID:47955<br>PDB ID: 9EEF	Kv1.3 G427H with NB1.3<br>EMDB ID:48072<br>PDB ID: 9EI0	Kv1.3 G427H Pore domain focused map<br>EMDB ID:47962<br>-
**Data collection and processing**							
Magnification	105000x	105000x	105000x	105000x	105000x	105000x	105000x
Voltage (kV)	300	300	300	300	300	300	300
Electron exposure (e–/Å^2)	58.58	52.16	52.16	51.8	50	51.6	50
Defocus range (μm)	-0.2 to -2.3	-0.4 to -2.4	-0.4 to -2.4	-0.8 to -2.4	-0.8 to -2.0	-0.8 to -2.0	-0.8 to -2.0
Pixel size (Å)	0.4125	0.4125	0.4125	0.4125	0.415	0.415	0.415
Symmetry imposed	C4	C4	C4	C4	C4	C4	C4
Initial particle images (no.)	2,352,524	2,423,268	2,423,268	4,971,787	2,763,104	5,391,638	2,763,104
Final particle images (no.)	122,877	115,627	109,882	102,939	139,210	259,382	139,210
Map resolution (Å)	2.97	3.06	3.03	3.18	3.56	2.91	3.05
FSC threshold	0.143	0.143	0.143	0.143	0.143	0.143	0.143
Map resolution range (Å)	1.77 - 5.88	1.79 - 6.38	1.81 - 5.98	2.06 - 6.94	2.27 - 6.87	1.78 - 5.91	1.80 - 3.33
**Refinement**							
Initial model used (PDB code)	7SSX	8DFL	8DFL	7SSX	7SSX	8DFL	
Model resolution (Å)	2.89	3.25	3.25	2.89	2.89	3.25	
FSC threshold	0.143	0.143	0.143	0.143	0.143	0.143	
Map sharpening *B* factor (Å^2)	-118.9	-109.3	-109	-121.2	-160.8	-129.6	
**Model composition**							
Non-hydrogen atoms	11152	15284	15284	11335	11400	15252	
Protein residues	1368	1908	1908	1384	1396	1892	
Ligands	4	4	4	2	4	4	
B factors (Å^2)							
Protein	62.72	77.93	89.43	84.44	61.77	84.31	
Ligand	1.51	15.21	18.2	6.49	59.81	16.3	
Water	-	-	-	24.93	-	-	
R.m.s. deviations							
Bond lengths (Å)	0.008	0.008	0.007	0.004	0.012	0.007	
Bond angles (°)	1.085	1.133	1.121	0.557	1.137	0.776	
Validation							
MolProbity score	1.75	2.07	2.03	1.65	2.01	1.81	
Clashscore	6.38	9.33	8.06	6.15	9.16	5.22	
Poor rotamers (%)	1.97	2.49	2.43	2.6	1.93	1.94	
Ramachandran plot							
Favored (%)	97.02	95.95	95.74	98.53	95.63	95.48	
Allowed (%)	2.98	4.05	4.26	1.47	4.37	4.52	
Disallowed (%)	0	0	0	0	0	0	

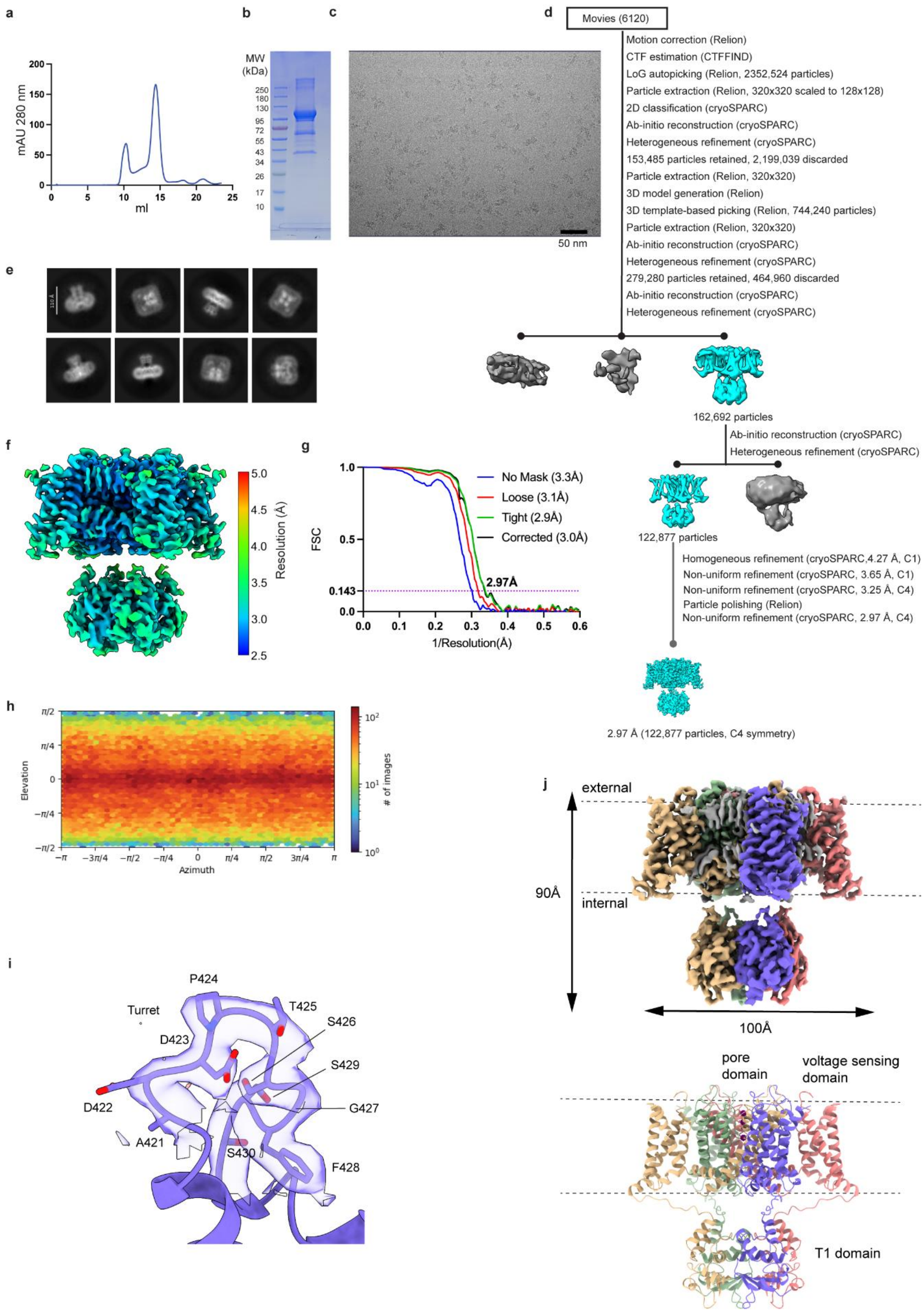
a
mAU 280 nm
ml
b
MW (kDa)
c
50 nm
d
Movies (6120)
Motion correction (Relion)
CTF estimation (CTFFIND)
LoG autopicking (Relion, 2352,524 particles)
Particle extraction (Relion, 320x320 scaled to 128x128)
2D classification (cryoSPARC)
Ab-initio reconstruction (cryoSPARC)
Heterogeneous refinement (cryoSPARC)
153,485 particles retained, 2,199,039 discarded
Particle extraction (Relion, 320x320)
3D model generation (Relion)
3D template-based picking (Relion, 744,240 particles)
Particle extraction (Relion, 320x320)
Ab-initio reconstruction (cryoSPARC)
Heterogeneous refinement (cryoSPARC)
279,280 particles retained, 464,960 discarded
Ab-initio reconstruction (cryoSPARC)
Heterogeneous refinement (cryoSPARC)
162,692 particles
Ab-initio reconstruction (cryoSPARC)
Heterogeneous refinement (cryoSPARC)
122,877 particles
Homogeneous refinement (cryoSPARC,4.27 Å, C1)
Non-uniform refinement (cryoSPARC, 3.65 Å, C1)
Non-uniform refinement (cryoSPARC, 3.25 Å, C4)
Particle polishing (Relion)
Non-uniform refinement (cryoSPARC, 2.97 Å, C4)
2.97 Å (122,877 particles, C4 symmetry)
e
f
Resolution (Å)
g
FSC
No Mask (3.3Å)
Loose (3.1Å)
Tight (2.9Å)
Corrected (3.0Å)
2.97Å
1/Resolution(Å)
h
Elevation
Azimuth
# of images
i
Turret
P424
T425
S426
D423
S429
D422
G427
A421
S430
F428
j
external
internal
90Å
100Å
pore domain
voltage sensing domain
T1 domain

← Fig. S3| Cryo-EM imaging for hKv1.3 H451V.

a) Superose 6 Increase (10/300 GL) size-exclusion chromatogram and **b**) SDS-PAGE for the purified hKv1.3 H451V mutant. **c**) Representative cryoEM image of hKv1.3 H451V mutant. **d**) Data processing workflow for hKv1.3 H451V mutant. Cryo-EM density maps are color-coded as cyan or gray according to whether they are retained or discarded, respectively. **e**) 2D class averages of hKv1.3 H451V mutant. **f**) Local resolution heat map of hKv1.3 H451V mutant. **g**) Fourier shell correlation (FSC) curves obtained from cryoSPARC with different masks. **h**) Angular distribution plot for particles in the reconstruction as generated by cryoSPARC. **i**) Cryo-EM map and model of the turret region of hKv1.3 H451V. **j**) Cryo-EM map of hKv1.3 H451V mutant in C4 symmetry with different subunits coloured (top) and model of hKv1.3 H451V with different domains indicated (bottom).

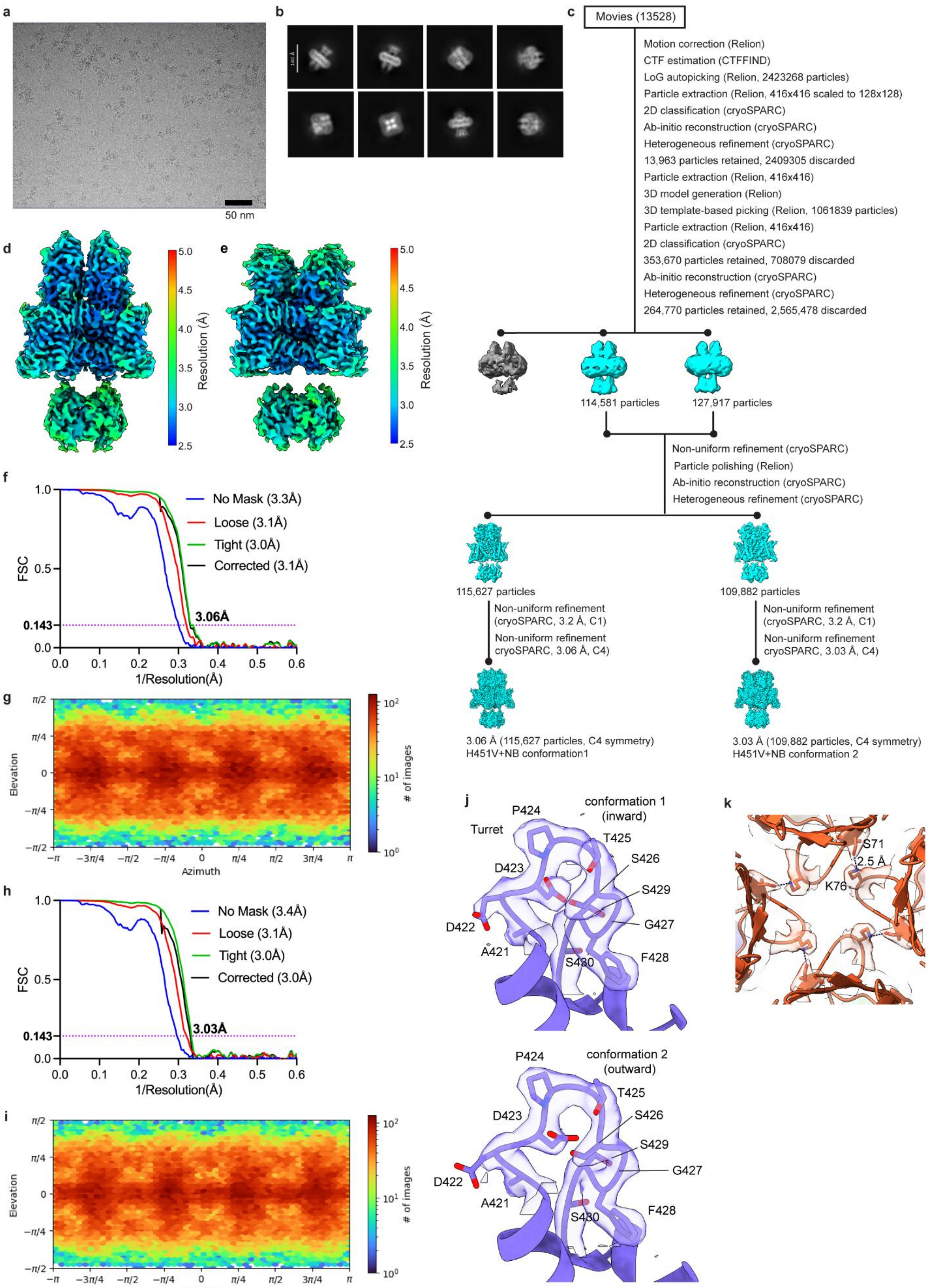
a
50 nm
b
c
Movies (13528)
Motion correction (Relion)
CTF estimation (CTFFIND)
LoG autopicking (Relion, 2423268 particles)
Particle extraction (Relion, 416x416 scaled to 128x128)
2D classification (cryoSPARC)
Ab-initio reconstruction (cryoSPARC)
Heterogeneous refinement (cryoSPARC)
13,963 particles retained, 2409305 discarded
Particle extraction (Relion, 416x416)
3D model generation (Relion)
3D template-based picking (Relion, 1061839 particles)
Particle extraction (Relion, 416x416)
2D classification (cryoSPARC)
353,670 particles retained, 708079 discarded
Ab-initio reconstruction (cryoSPARC)
Heterogeneous refinement (cryoSPARC)
264,770 particles retained, 2,565,478 discarded
114,581 particles
127,917 particles
Non-uniform refinement (cryoSPARC)
Particle polishing (Relion)
Ab-initio reconstruction (cryoSPARC)
Heterogeneous refinement (cryoSPARC)
115,627 particles
109,882 particles
Non-uniform refinement (cryoSPARC, 3.2 Å, C1)
Non-uniform refinement cryoSPARC, 3.06 Å, C4)
Non-uniform refinement (cryoSPARC, 3.2 Å, C1)
Non-uniform refinement cryoSPARC, 3.03 Å, C4)
3.06 Å (115,627 particles, C4 symmetry) H451V+NB conformation1
3.03 Å (109,882 particles, C4 symmetry) H451V+NB conformation 2
d
e
Resolution (Å)
5.0
4.5
4.0
3.5
3.0
2.5
f
FSC
No Mask (3.3Å)
Loose (3.1Å)
Tight (3.0Å)
Corrected (3.1Å)
3.06Å
0.143
1/Resolution(Å)
g
Elevation
Azimuth
# of images
h
FSC
No Mask (3.4Å)
Loose (3.1Å)
Tight (3.0Å)
Corrected (3.0Å)
3.03Å
0.143
1/Resolution(Å)
i
Elevation
Azimuth
# of images
j
conformation 1 (inward)
Turret
P424
T425
S426
D423
S429
G427
D422
A421
S430
F428
conformation 2 (outward)
P424
T425
D423
S426
S429
G427
D422
A421
S430
F428
k
S71
2.5 Å
K76

← Fig. S4| Cryo-EM imaging for the H451V mutant of hKv1.3 with NB1.3 bound.

a) Representative cryoEM image of hKv1.3 H451V mutant in complex with NB1.3. **b**) 2D class averages of hKv1.3 H451V mutant in complex with NB1.3 **c**) Data processing workflow for hKv1.3 H451V mutant in complex with NB1.3. Cryo-EM density maps are color-coded as cyan or gray according to whether they are retained or discarded, respectively. **d**) Local resolution heat map of hKv1.3 H451V mutant in complex with NB1.3 conformation 1. **e**) Local resolution heat map of hKv1.3 H451V mutant in complex with NB1.3 conformation 2. **f**) Fourier shell correlation (FSC) curves obtained from cryoSPARC with different masks for hKv1.3 H451V mutant in complex with NB1.3 conformation1. **g**) Angular distribution plot for particles in the reconstruction as generated by cryoSPARC for hKv1.3 H451V mutant in complex with NB1.3 conformation 1. **h**) Fourier shell correlation (FSC) curves obtained from cryoSPARC with different masks for hKv1.3 H451V mutant in complex with NB1.3 conformation 2. **i**) Angular distribution plot for particles in the reconstruction as generated by cryoSPARC for hKv1.3 H451V mutant in complex with NB1.3 conformation 2. **j**) Cryo-EM density and models for the turret regions of H451V in complex with NB1.3 for both conformations. **k**) Cryo-EM map and structural model of NB1.3 bound to the external surface of hKv1.3 H451V in conformation 1 showing interactions between individual molecules of NB1.3.

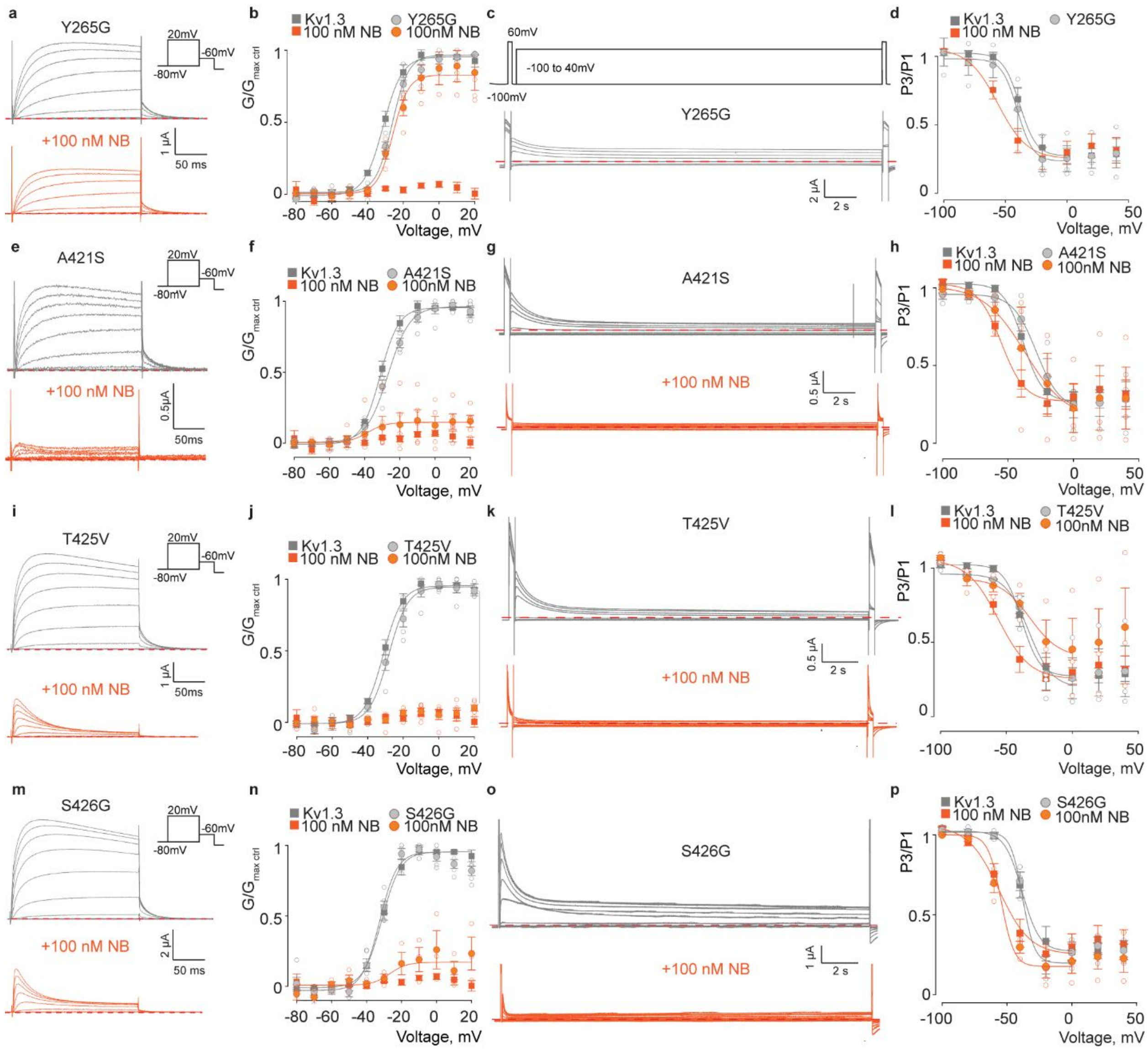

a
Y265G
20mV
-60mV
-80mV
+100 nM NB
1 μA
50 ms
b
Kv1.3
Y265G
100 nM NB
100nM NB
G/G max ctrl
Voltage, mV
c
60mV
-100 to 40mV
-100mV
Y265G
2 μA
2 s
d
Kv1.3
Y265G
100 nM NB
P3/P1
Voltage, mV
e
A421S
+100 nM NB
0.5μA
50ms
f
Kv1.3
A421S
100 nM NB
100nM NB
g
A421S
+100 nM NB
0.5 μA
2 s
h
Kv1.3
A421S
100 nM NB
100nM NB
i
T425V
+100 nM NB
1 μA
50ms
j
Kv1.3
T425V
100 nM NB
100nM NB
k
T425V
+100 nM NB
0.5 μA
2 s
l
Kv1.3
T425V
100 nM NB
100nM NB
m
S426G
+100 nM NB
2 μA
50 ms
n
Kv1.3
S426G
100 nM NB
100nM NB
o
S426G
+100 nM NB
1 μA
2 s
p
Kv1.3
S426G
100 nM NB
100nM NB

← **Fig. S5| Functional data for S1-S2 loop and turret mutants.**

a) Families of currents elicited by depolarization in the absence and presence of 100 nM NB1.3 for the hKv1.3 Y265G mutant. **b)** Plot of normalized conductance (G/G_{max}) obtained from tail current measurements for WT hKv1.3 (data from Fig. 1i,j) and the Y265G mutant in the absence and presence of 100 nM NB1.3. Smooth curves are Boltzmann fits with $V_{1/2}$ and z values of -26.3 mV and 5.6 for Y265F in control solution and -25.8 mV and 5.9 in the presence of NB1.3 (n = 4 in 3 independent experiments). Error bars represent S.E.M. **c)** Current traces obtained using a three-pulse protocol for the Y265G mutant in control solution. **d)** Plot of P3/P1 for WT hKv1.3 in the absence and presence of NB1.3 and Y265G mutant in control solution. Smooth curve is a Boltzmann fit with $V_{1/2}$ and z values of -40.3 mV and 4.1 for Y265G in control solution (n = 4 in 3 independent experiments). Error bars represent S.E.M. WT data are from Fig. 1g. **e)** Families of currents elicited by depolarization in the absence and presence of 100 nM NB1.3 for the A421S mutant. **f)** Plot of normalized conductance (G/G_{max}) obtained from tail current measurements for WT hKv1.3 and the A421S mutant in the absence and presence of 100 nM NB1.3. Smooth curves are Boltzmann fits with $V_{1/2}$ and z values of -26.7 mV and 4.2 for A421S in control solution and -38.3 mV and 5.1 in the presence of NB1.3 (n = 5 in 3 independent experiments). Error bars represent S.E.M. WT data are from Fig. 1j. **g)** Current traces obtained using a three-pulse protocol for A421S in the absence and presence of 100 nM NB1.3. **h)** Plot of P3/P1 for WT hKv1.3 and the A421S mutant in the absence and presence of 100 nM NB1.3. Smooth curves are Boltzmann fits with $V_{1/2}$ and z values of -28.4 mV and 2.9 for A421S in control solution and -36.9 mV and 1.6 in the presence of NB1.3 (n = 3 in 3 independent experiments). Error bars represent S.E.M. WT data are from Fig. 1g. **i)** Families of currents elicited by depolarization in the absence and presence of 100 nM NB1.3 for the T425V mutant. **j)** Plot of normalized conductance (G/G_{max}) obtained from tail current measurements for WT hKv1.3 and the T425V mutant in the absence and presence of 100 nM NB1.3. Smooth curves are Boltzmann fits with $V_{1/2}$ and z values of -28.3 mV and 4.8 for T425V in control solution and in the presence of NB1.3 currents were too small for reliable fitting (n = 5 in 4 independent experiments). Error bars represent S.E.M. WT data are from Fig. 1j. **k)** Current traces obtained using a three-pulse protocol for T425V in the absence and presence of 100 nM NB1.3. **l)** Plot of P3/P1 for WT hKv1.3 and the T425V mutant in the absence and presence of 100 nM NB1.3. Smooth curves are Boltzmann fits with $V_{1/2}$ and z values of -33.2 mV and 2.9 for T425V in control solution and -32.2 mV and 2.3 in the presence of NB1.3 (n = 3 in 3 independent experiments). Error bars represent S.E.M. WT data are from Fig. 1g. **m)** Families of currents elicited by depolarization in the absence and presence of 100 nM NB1.3 for the S426G mutant. **n)** Plot of normalized conductance (G/G_{max}) obtained from tail current measurements for WT hKv1.3 and the S426G mutant in the absence and presence of 100 nM NB1.3. Smooth curves are Boltzmann fits with $V_{1/2}$ and z values of -32.6 mV and 5.1 for S426G in control solution (n = 5 in 4 independent experiments) and -26.8 mV and 5.3 in the presence of NB1.3 (n = 3 in 3 independent experiments). Error bars represent S.E.M. WT data are from Fig. 1j. **o)** Current traces obtained using a three-pulse protocol for S426G in the absence and presence of 100 nM NB1.3. **p)** Plot of P3/P1 for WT hKv1.3 and the S426G mutant in the absence and presence of 100 nM NB1.3. Smooth curves are Boltzmann fits with $V_{1/2}$ and z values of -37.9 mV and 5.6 for S426G in control solution and -54.5 mV and 5.1 in the presence of NB1.3 (n = 3 in 3 independent experiments). Error bars represent S.E.M. WT data are from Fig. 1g.

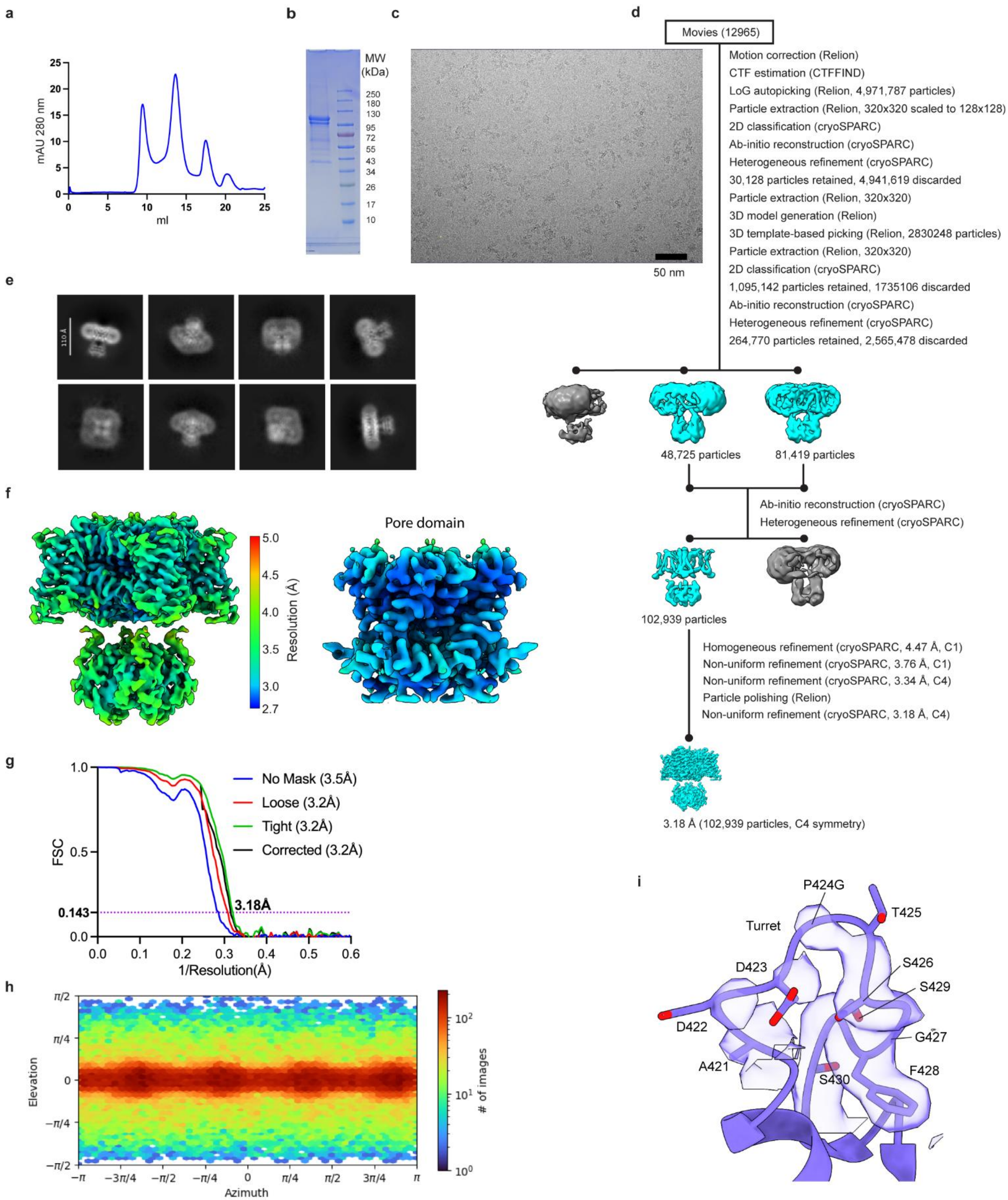
a
mAU 280 nm
ml
b
MW (kDa)
250
180
130
95
72
55
43
34
26
17
10
c
50 nm
d
Movies (12965)
Motion correction (Relion)
CTF estimation (CTFFIND)
LoG autopicking (Relion, 4,971,787 particles)
Particle extraction (Relion, 320x320 scaled to 128x128)
2D classification (cryoSPARC)
Ab-initio reconstruction (cryoSPARC)
Heterogeneous refinement (cryoSPARC)
30,128 particles retained, 4,941,619 discarded
Particle extraction (Relion, 320x320)
3D model generation (Relion)
3D template-based picking (Relion, 2830248 particles)
Particle extraction (Relion, 320x320)
2D classification (cryoSPARC)
1,095,142 particles retained, 1735106 discarded
Ab-initio reconstruction (cryoSPARC)
Heterogeneous refinement (cryoSPARC)
264,770 particles retained, 2,565,478 discarded
48,725 particles
81,419 particles
Ab-initio reconstruction (cryoSPARC)
Heterogeneous refinement (cryoSPARC)
102,939 particles
Homogeneous refinement (cryoSPARC, 4.47 Å, C1)
Non-uniform refinement (cryoSPARC, 3.76 Å, C1)
Non-uniform refinement (cryoSPARC, 3.34 Å, C4)
Particle polishing (Relion)
Non-uniform refinement (cryoSPARC, 3.18 Å, C4)
3.18 Å (102,939 particles, C4 symmetry)
e
110 Å
f
Resolution (Å)
Pore domain
g
FSC
No Mask (3.5Å)
Loose (3.2Å)
Tight (3.2Å)
Corrected (3.2Å)
3.18Å
1/Resolution(Å)
h
Elevation
Azimuth
# of images
i
P424G
T425
Turret
S426
S429
D423
D422
G427
A421
S430
F428

← Fig. S6| Cryo-EM imaging of the P424G mutant of hKv1.3.

a) Superose 6 Increase (10/300 GL) size-exclusion chromatogram and **b**) SDS-PAGE for the purified hKv1.3 P424G mutant. **c**) Representative cryoEM image of Kv1.3 P424G mutant. **d**) Data processing workflow for hKv1.3 P424G mutant. Cryo-EM density maps are color-coded as cyan or gray according to whether they are retained or discarded, respectively. **e**) 2D class averages of hKv1.3 P424G mutant. **f**) Local resolution heat map of hKv1.3 P424G mutant (left) and pore domain (right). **g**) Fourier shell correlation (FSC) curves obtained from cryoSPARC with different masks. **h**) Angular distribution plot for particles in the reconstruction as generated by cryoSPARC. **i**) Cryo-EM density and structural models of turret loop of P424G mutant of hKv1.3 displaying weak density for the region between D423-T425.

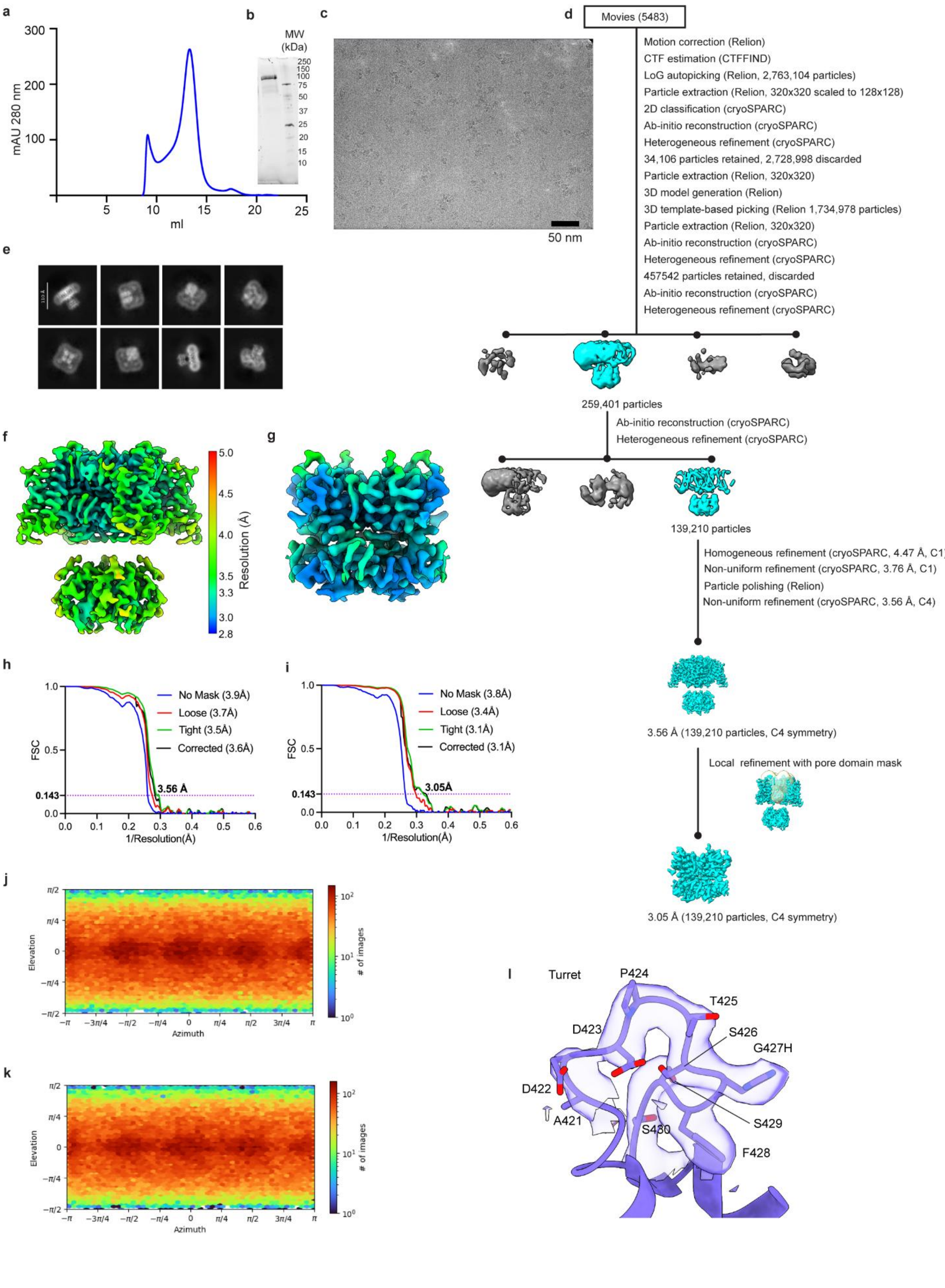

a
mAU 280 nm
ml
b
MW (kDa)
250
150
100
75
50
37
25
20
15
10
c
50 nm
d
Movies (5483)
Motion correction (Relion)
CTF estimation (CTFFIND)
LoG autopicking (Relion, 2,763,104 particles)
Particle extraction (Relion, 320x320 scaled to 128x128)
2D classification (cryoSPARC)
Ab-initio reconstruction (cryoSPARC)
Heterogeneous refinement (cryoSPARC)
34,106 particles retained, 2,728,998 discarded
Particle extraction (Relion, 320x320)
3D model generation (Relion)
3D template-based picking (Relion 1,734,978 particles)
Particle extraction (Relion, 320x320)
Ab-initio reconstruction (cryoSPARC)
Heterogeneous refinement (cryoSPARC)
457542 particles retained, discarded
Ab-initio reconstruction (cryoSPARC)
Heterogeneous refinement (cryoSPARC)
259,401 particles
Ab-initio reconstruction (cryoSPARC)
Heterogeneous refinement (cryoSPARC)
139,210 particles
Homogeneous refinement (cryoSPARC, 4.47 Å, C1)
Non-uniform refinement (cryoSPARC, 3.76 Å, C1)
Particle polishing (Relion)
Non-uniform refinement (cryoSPARC, 3.56 Å, C4)
3.56 Å (139,210 particles, C4 symmetry)
Local refinement with pore domain mask
3.05 Å (139,210 particles, C4 symmetry)
e
f
Resolution (Å)
g
h
FSC
No Mask (3.9Å)
Loose (3.7Å)
Tight (3.5Å)
Corrected (3.6Å)
3.56 Å
1/Resolution(Å)
i
FSC
No Mask (3.8Å)
Loose (3.4Å)
Tight (3.1Å)
Corrected (3.1Å)
3.05Å
1/Resolution(Å)
j
Elevation
Azimuth
# of images
k
Elevation
Azimuth
# of images
l
Turret
P424
T425
D423
S426
G427H
D422
A421
S430
S429
F428

← Fig. S7| Cryo-EM imaging for the G427H mutant of hKv1.3.

a) Superose 6 Increase (10/300 GL) size-exclusion chromatogram and **b**) SDS-PAGE for the purified hKv1.3 G427H mutant. **c**) Representative cryo-EM image of hKv1.3 G427H mutant. **d**) Data processing workflow for hKv1.3 G427H mutant. Cryo-EM density maps are color-coded as cyan or gray according to whether they are retained or discarded, respectively. **e**) 2D class averages of hKv1.3 G427H mutant. **f,g**) Local resolution heat map for the entire channel (f) or only the pore domain (g) for the hKv1.3 G427H mutant. **h,i**) Fourier shell correlation (FSC) curves obtained from cryoSPARC with different masks for the entire channel (h) or the pore domain (i). **j,k**) Angular distribution plot for particles in the reconstruction as generated by cryoSPARC for the entire channel (h) or the pore domain (i). **l**) Cryo-EM map and structural model of the turret region of hKv1.3 G427H.

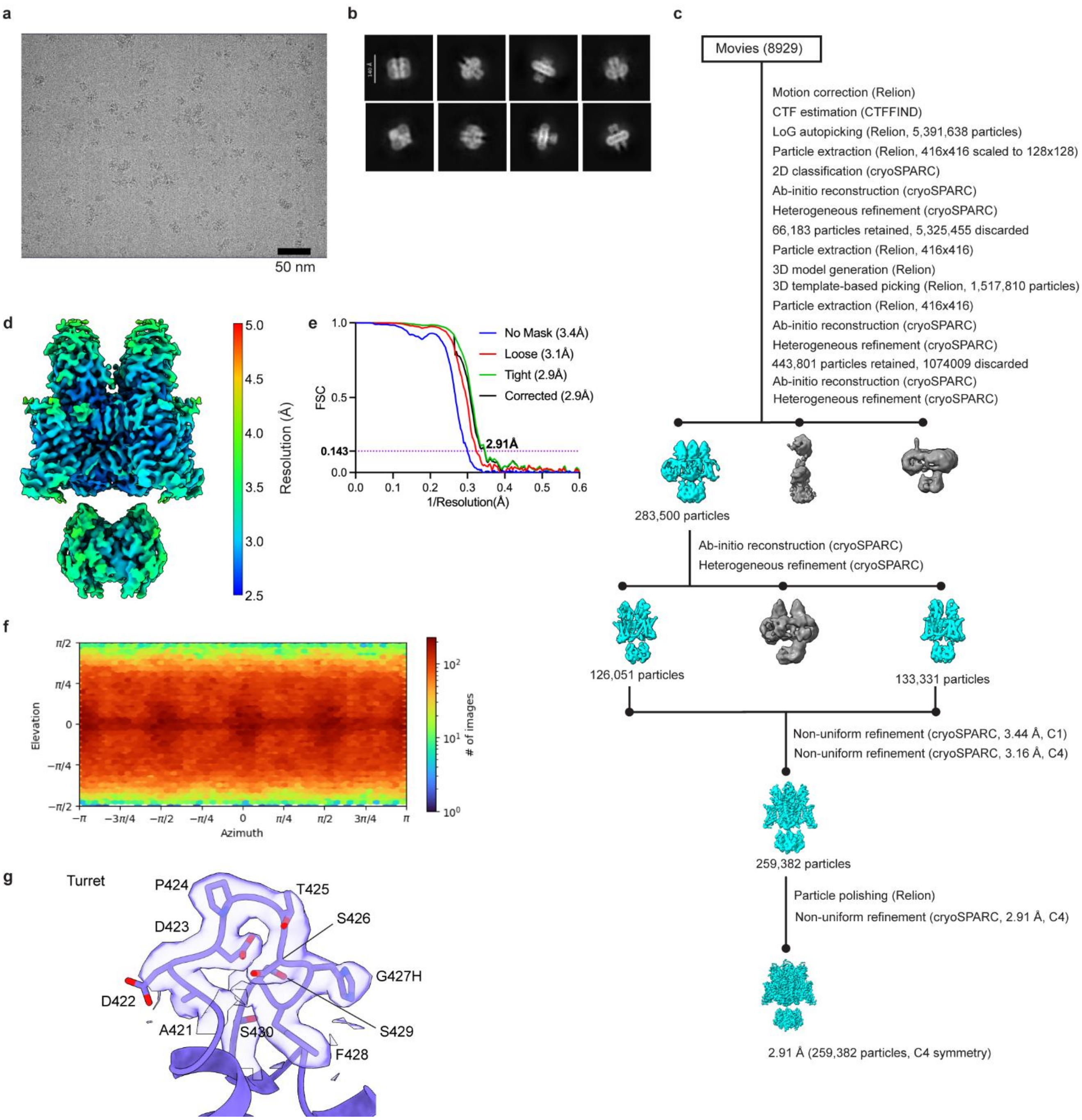
a
50 nm
b
c
Movies (8929)
Motion correction (Relion)
CTF estimation (CTFFIND)
LoG autopicking (Relion, 5,391,638 particles)
Particle extraction (Relion, 416x416 scaled to 128x128)
2D classification (cryoSPARC)
Ab-initio reconstruction (cryoSPARC)
Heterogeneous refinement (cryoSPARC)
66,183 particles retained, 5,325,455 discarded
Particle extraction (Relion, 416x416)
3D model generation (Relion)
3D template-based picking (Relion, 1,517,810 particles)
Particle extraction (Relion, 416x416)
Ab-initio reconstruction (cryoSPARC)
Heterogeneous refinement (cryoSPARC)
443,801 particles retained, 1074009 discarded
Ab-initio reconstruction (cryoSPARC)
Heterogeneous refinement (cryoSPARC)
283,500 particles
Ab-initio reconstruction (cryoSPARC)
Heterogeneous refinement (cryoSPARC)
126,051 particles
133,331 particles
Non-uniform refinement (cryoSPARC, 3.44 Å, C1)
Non-uniform refinement (cryoSPARC, 3.16 Å, C4)
259,382 particles
Particle polishing (Relion)
Non-uniform refinement (cryoSPARC, 2.91 Å, C4)
2.91 Å (259,382 particles, C4 symmetry)
d
Resolution (Å)
5.0
4.5
4.0
3.5
3.0
2.5
e
FSC
1/Resolution(Å)
No Mask (3.4Å)
Loose (3.1Å)
Tight (2.9Å)
Corrected (2.9Å)
2.91Å
0.143
f
Elevation
Azimuth
# of images
g
Turret
P424
T425
D423
S426
G427H
D422
A421
S430
S429
F428

← Fig. S8| Cryo-EM imaging for the G427H mutant of hKv1.3 with NB1.3 bound.

a) Representative cryo-EM image of hKv1.3 G427H mutant in complex with NB1.3. **b**) 2D class averages of hKv1.3 G427H mutant in complex with NB1.3 **c**) Data processing workflow for hKv1.3 G427H mutant in complex with NB1.3. Cryo-EM density maps are color-coded as cyan or gray according to whether they are retained or discarded, respectively. **d**) Local resolution heat map. **e**) Fourier shell correlation (FSC) curves obtained from cryoSPARC with different masks for hKv1.3 G427H mutant in complex with NB1.3. **f**) Angular distribution plot for particles in the reconstruction as generated by cryoSPARC. **g**) Cryo-EM map and structural model of the turret region of hKv1.3 G427H in complex with NB1.3.

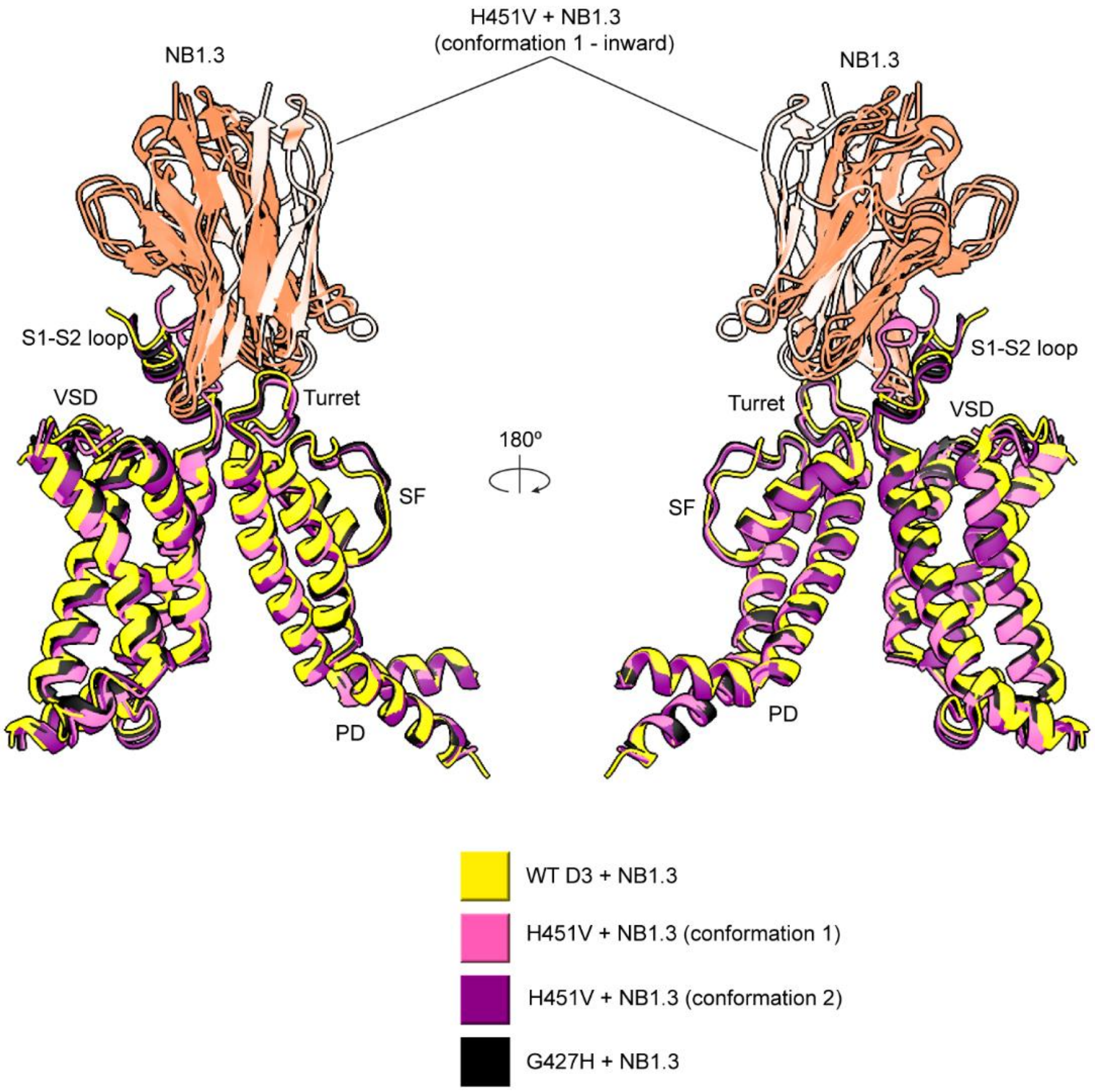

Fig. S9| Superimposition of models for WT and mutant hKv1.3 with NB1.3 bound.

Interaction interfaces between NB1.3 and the S1-S2 loop and turret are similar for all structures and the NB with lighter shading is for the inward leaning conformation of H451V + NB1.3 (conformation 1).

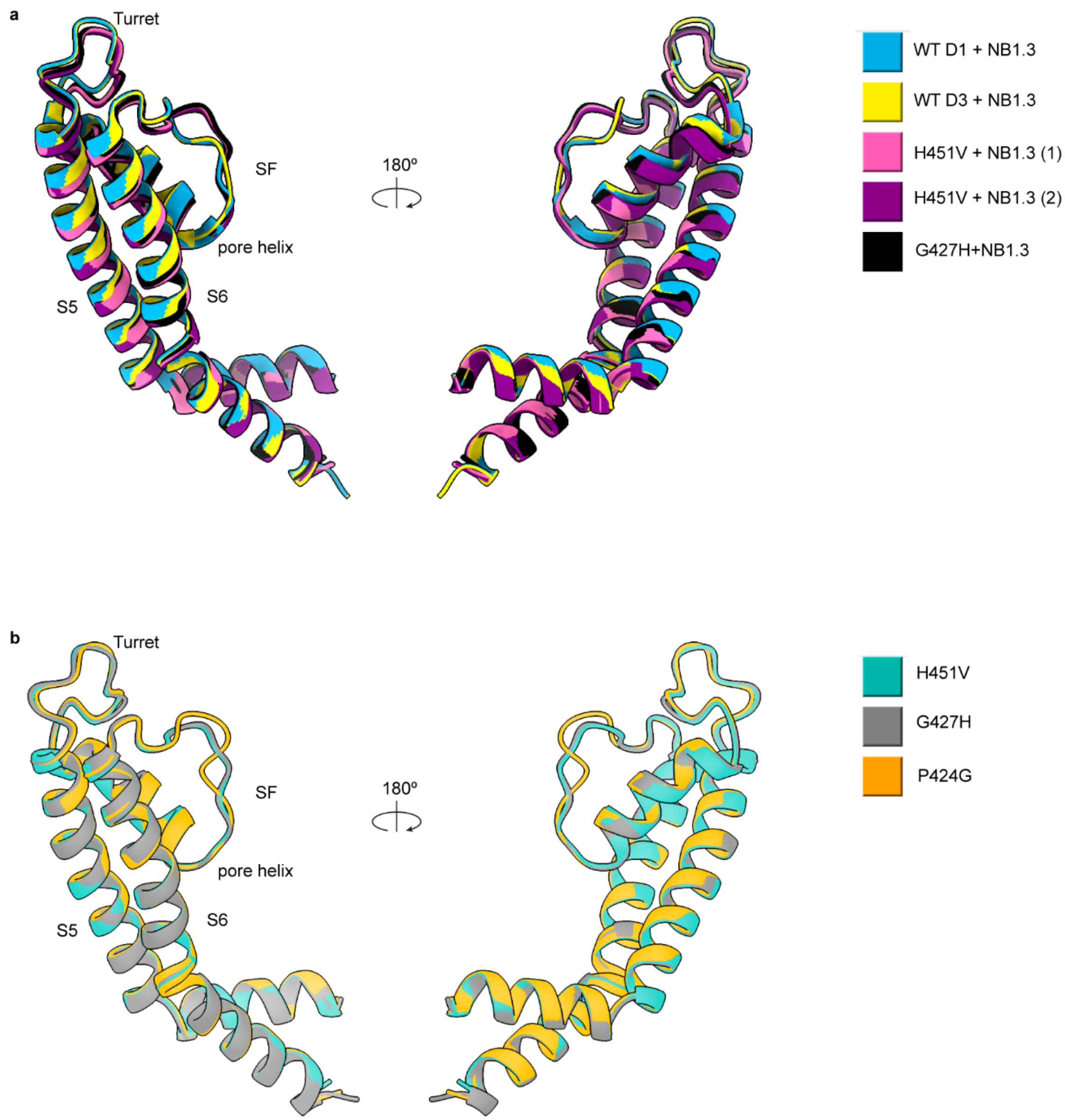

Fig. S10| Superimposition of models for the individual subunits contributing to the pore domains of WT and mutant hKv1.3 channels

Structures of hKv1.3 subunits are shown with NB1.3 bound (a) or under control conditions (b).

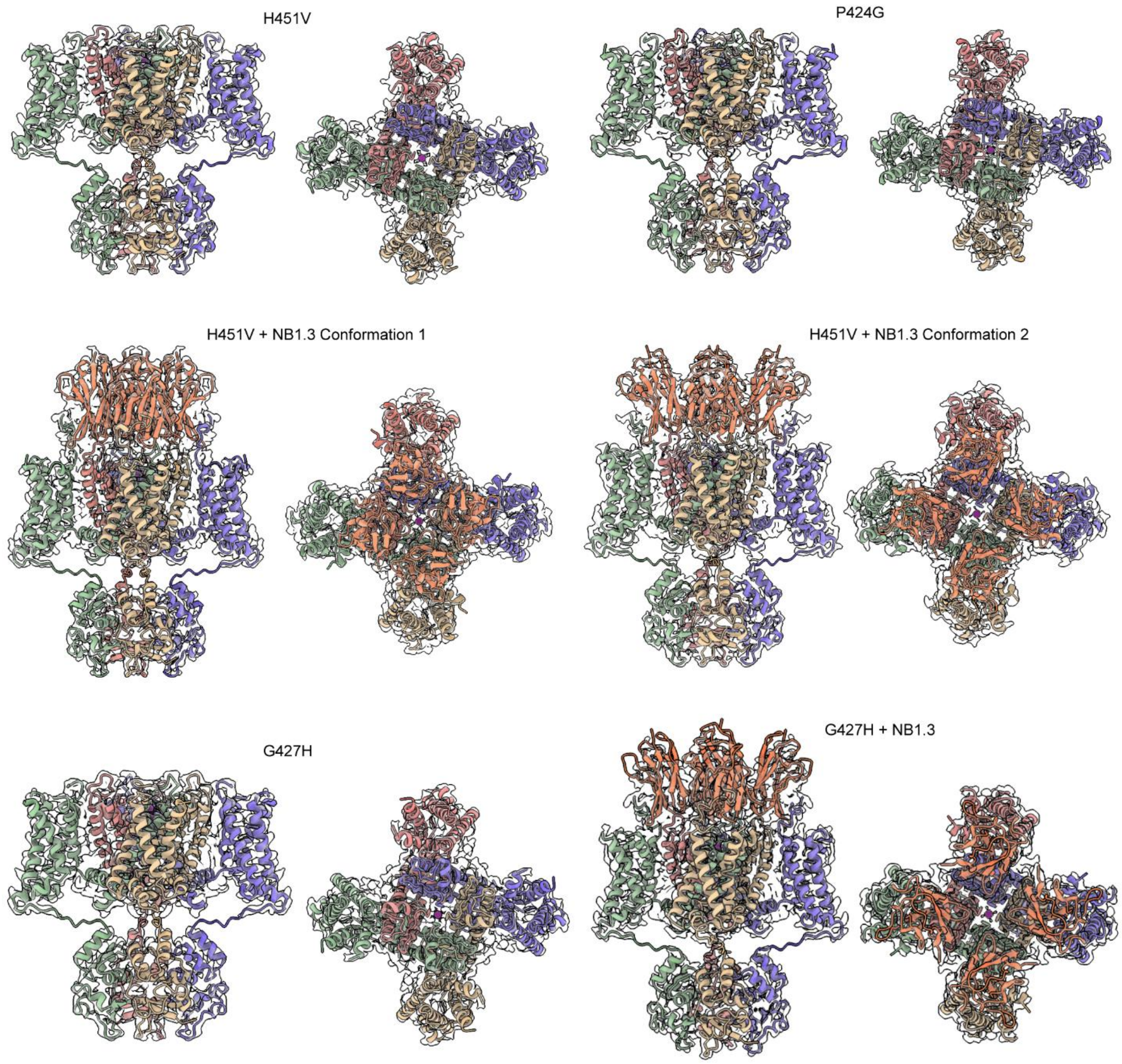

Fig. S11| Maps and models of all the structures of hKv1.3 mutants from the present study.

Contour levels at σ = 3.0 are 0.2 for H451V, 0.2 for P424G, 0.25 for H451V with NB1.3 conformation 1, 0.2 for H451V with NB1.3 conformation 2, 0.15 for G427H and 0.2 for G427H with NB1.3.

Movie S1| Morph between the structure of hKv1.3 H451V and WT D1 and D3 with NB1.3 bound.
