# Supplementary material for: Nanobody regulation of C-type inactivation in Kv1.3 channels
Source: Nat Commun. 2026 Aug 4;17:9379. doi: 10.1038/s41467-026-74630-8 (PMC13534573; doi:10.1038/s41467-026-74630-8)
Supplement: Supplementary file 2 [file 41467_2026_74630_MOESM2_ESM.pdf]

## Description of Additional Supplementary Files

**File Name:** Supplementary Movie 1

**Description:** Morph between the structures of hKv1.3 H451V and WT D1 and D3 with NB1.3 bound.
