# Supplementary material for: Nanobody regulation of C-type inactivation in Kv1.3 channels
Source: Nat Commun. 2026 Aug 4;17:9379. doi: 10.1038/s41467-026-74630-8 (PMC13534573; doi:10.1038/s41467-026-74630-8)
Supplement: Supplementary file 3 [file 41467_2026_74630_MOESM4_ESM.pdf]

nature portfolio

Corresponding author(s): Ana I. Fernández-Mariño
Last updated by author(s): May 17, 2026

Reporting Summary

Nature Portfolio wishes to improve the reproducibility of the work that we publish. This form provides structure for consistency and transparency in reporting. For further information on Nature Portfolio policies, see our Editorial Policies and the Editorial Policy Checklist.

Statistics

For all statistical analyses, confirm that the following items are present in the figure legend, table legend, main text, or Methods section.

n/a	Confirmed	
☐	☒	The exact sample size (*n*) for each experimental group/condition, given as a discrete number and unit of measurement
☐	☒	A statement on whether measurements were taken from distinct samples or whether the same sample was measured repeatedly
☒	☐	The statistical test(s) used AND whether they are one- or two-sided<br>*Only common tests should be described solely by name; describe more complex techniques in the Methods section.*
☒	☐	A description of all covariates tested
☒	☐	A description of any assumptions or corrections, such as tests of normality and adjustment for multiple comparisons
☐	☒	A full description of the statistical parameters including central tendency (e.g. means) or other basic estimates (e.g. regression coefficient) AND variation (e.g. standard deviation) or associated estimates of uncertainty (e.g. confidence intervals)
☒	☐	For null hypothesis testing, the test statistic (e.g. *F*, *t*, *r*) with confidence intervals, effect sizes, degrees of freedom and *P* value noted<br>*Give P values as exact values whenever suitable.*
☒	☐	For Bayesian analysis, information on the choice of priors and Markov chain Monte Carlo settings
☒	☐	For hierarchical and complex designs, identification of the appropriate level for tests and full reporting of outcomes
☒	☐	Estimates of effect sizes (e.g. Cohen's *d*, Pearson's *r*), indicating how they were calculated

Our web collection on statistics for biologists contains articles on many of the points above.

Software and code

Policy information about availability of computer code

Data collection	Leginon and SerialEM 3.8.1 for cryo-EM and pClamp 10.7 for electrophysiology
Data analysis	RELION 3.0 and 4.0, cryoSPARC v4.5.3, CTFFIND4 for cryo-EM. Phenix v1.21.2 and Coot 0.9.8.92 for model building, UCSF Chimera 1.17.3 and ChimeraX 1.9 for displaying maps and models in figures. pClamp 10.7 and Origin 2023b for electrophysiology.

For manuscripts utilizing custom algorithms or software that are central to the research but not yet described in published literature, software must be made available to editors and reviewers. We strongly encourage code deposition in a community repository (e.g. GitHub). See the Nature Portfolio guidelines for submitting code & software for further information.

Data

Policy information about availability of data

All manuscripts must include a data availability statement. This statement should provide the following information, where applicable:

- Accession codes, unique identifiers, or web links for publicly available datasets
- A description of any restrictions on data availability
- For clinical datasets or third party data, please ensure that the statement adheres to our policy

All data needed to evaluate the conclusions in the paper are present in the paper and/or the Supplementary Materials. Models of hKv1.3 mutant proteins have been deposited in the Protein Data Bank with accession codes 9CLA for hKv1.3 H451V mutant, 9CON for hKv1.3 H451V mutant in complex with NB1.3 conformation1, 9CTY for hKv1.3 H451V mutant in complex with NB1.3 conformation2, 9CLV for hKv1.3 P424G mutant, 9EEF for hKv1.3 G427H mutant and 9EI0 for hKv1.3 G427 mutant in complex with NB1.3. Maps of hKv1.3 mutants have been deposited in the Electron Microscopy Data Bank (EMDB) under accession codes

EMD-45668 for hKv1.3 H451V mutant, EMD-45786 for hKv1.3 H451V mutant in complex with NB1.3 conformation1, EMD-45922- hKv1.3 H451V mutant in complex with NB1.3 conformation2, EMD-45731 for hKv1.3 P424G mutant, EMD-47955 for hKv1.3 G427H mutant, EMD-47962 for hKv1.3 G427H pore domain, EMD-48072 for hKv1.3 G427H in complex with NB1.3.

Research involving human participants, their data, or biological material

Policy information about studies with human participants or human data. See also policy information about sex, gender (identity/presentation), and sexual orientation and race, ethnicity and racism.

Reporting on sex and gender	n/a
Reporting on race, ethnicity, or other socially relevant groupings	n/a
Population characteristics	n/a
Recruitment	n/a
Ethics oversight	n/a

Note that full information on the approval of the study protocol must also be provided in the manuscript.

Field-specific reporting

Please select the one below that is the best fit for your research. If you are not sure, read the appropriate sections before making your selection.

[x] Life sciences   [ ] Behavioural & social sciences   [ ] Ecological, evolutionary & environmental sciences

For a reference copy of the document with all sections, see nature.com/documents/nr-reporting-summary-flat.pdf

Life sciences study design

All studies must disclose on these points even when the disclosure is negative.

Sample size	Statistical methods were not used to determine sample size. Sample size for cryo-EM studies was determined by availability of microscope time and to ensure we obtain sufficient resolution for model building. Sample size for electrophysiological studies was determined empirically by comparing individual measurements with population data obtained under differing conditions until convincing differences or lack thereof were evident.
Data exclusions	For electrophysiological experiments, exploratory experiments were undertaken with varying ionic conditions and voltage-clamp protocols to define ideal conditions for measurements reported in this study. Although these preliminary experiments are consistent with the results we report, they were not included in our analysis due to varying experimental conditions. Once ideal conditions were identified, electrophysiological data were collected for control and mutant constructs until convincing trends in population datasets were obtained. Individual cells were also excluded if cells exhibited excessive initial leak currents at the holding voltage (>0.5 µA), if currents arising from expressed channels were too small (<0.5 µA), making it difficult to distinguish the activity of expressed channels from endogenous channels, or if currents arising from expressed channels were too large, resulting in substantial voltage errors or changes in the concentration of ions in either intracellular or extracellular solutions.
Replication	Information on sample size is provided in figure legends throughout the manuscript.
Randomization	Randomization was not used in this study, because these experiments did not involve allocating discrete samples to experimental groups.
Blinding	Blinding was not used in this study, because the knowledge of the sample doesn't affect the measurement of the datasets.

Reporting for specific materials, systems and methods

We require information from authors about some types of materials, experimental systems and methods used in many studies. Here, indicate whether each material, system or method listed is relevant to your study. If you are not sure if a list item applies to your research, read the appropriate section before selecting a response.

Materials & experimental systems

n/a	Involved in the study
☐	☒ Antibodies
☐	☒ Eukaryotic cell lines
☒	☐ Palaeontology and archaeology
☐	☒ Animals and other organisms
☒	☐ Clinical data
☒	☐ Dual use research of concern
☒	☐ Plants

Methods

n/a	Involved in the study
☒	☐ ChIP-seq
☒	☐ Flow cytometry
☒	☐ MRI-based neuroimaging

Antibodies

Antibodies used: A019400G09 nanobody was recombinantly expressed in E.coli Bl-21DE3. Nanobody Sequence was obtained from the patent (https://patents.google.com/patent/CA2951443A1/en)

Validation: Nanobody binding was validated by Electrophysiology and cryo-EM

Eukaryotic cell lines

Policy information about cell lines and Sex and Gender in Research

Cell line source(s): Sf9, EXPI 293F cells were originally obtained from Thermo Fischer and tsa201 from Sigma-Aldrich.

Authentication: Cell lines used were not authenticated.

Mycoplasma contamination: Mycoplasma contamination was tested and found to be negative

Commonly misidentified lines (See ICLAC register): commonly misidentified cell lines were not used in this study

Animals and other research organisms

Policy information about studies involving animals; ARRIVE guidelines recommended for reporting animal research, and Sex and Gender in Research

Laboratory animals: female Xenopus laevis frogs, 1-2 years of age, obtained from Xenopus I

Wild animals: no wild animals were used in the study

Reporting on sex: Female

Field-collected samples: no field collected samples were used in the study

Ethics oversight: The animal care and experimental procedures were performed in accordance with the Guide for the Care and Use of Laboratory Animals and were approved by the Animal Care and Use Committee of the National Institute of Neurological Disorders and Stroke (animal protocol number 1253).

Note that full information on the approval of the study protocol must also be provided in the manuscript.

Plants

Seed stocks: n/a

Novel plant genotypes: n/a

Authentication: n/a
