# Supplementary material for: Nanobody regulation of C-type inactivation in Kv1.3 channels
Source: Nat Commun. 2026 Aug 4;17:9379. doi: 10.1038/s41467-026-74630-8 (PMC13534573; doi:10.1038/s41467-026-74630-8)
Supplement: Supplementary file 4 [file 41467_2026_74630_MOESM5_ESM.pdf]

nature portfolio

Peer Review File

Nanobody regulation of C-type inactivation in Kv1.3 channels

Corresponding Author: Dr Ana Fernández-Mariño

This file contains all reviewer reports in order by version, followed by all author rebuttals in order by version.

Version 0:

Reviewer comments:

Reviewer #1

(Remarks to the Author)
The authors of this study previously reported the cryo-EM structures of Kv1.3 in the apo-state and in complex with the NB1.3 nanobody and an ShK-knotbody. However, the previously resolved apo structure was in the C-type inactivated state with a dilated selectivity filter and the field is therefore missing structures of open, conducting states of Kv1.3 and insight into the mechanism of action of the NB1.3 nanobody, which is binding to the turret of the channel and inhibits the channel by speeding up inactivation.

In the current study the authors use a combination of mutagenesis, electrophysiology and cryo-EM to characterize and then capture several mutants with reduced C-type inactivation (H451V, G427H) in the open conducting state with a fully occupied selectivity filter. The authors further map the interface between the nanobody and the channel turret and the S1-S2 loop and again obtain the structure of a key mutant (P424G) to obtain deeper insight into how the nanobody induces C-type inactivation by modulating the turret and eventually dilating the selectivity filter through modification of a network of hydrophobic residues.

The work is important in providing insights into the mechanisms of C-type inactivation in Kv1 family channels and possibly enabling design of nanobodies targeting tetrameric cation channels.

As a reviewer I have several suggestions for the authors:

1) Would you dare to make a little movie of morphed structures showing how the nanobody induces C-type inactivation? If you do not think this is possible at this point, please rebut.

2) Please clarify if the two nanobody conformations observed with the H451V mutant with nanobodies either leaning inward or outward were also observed in the previously captured structure of the nanobody with the WT channels. Fig 2 seems to suggest that the previous structure only showed the inward conformation. Please specify the confirmation and maybe speculate if the nanobody progresses from outward to inward when it inhibits the WT channel.

3) Please also specify if there are any direct contacts between the four nanobodies in the inward confirmation. Again, Figure 2 seems to suggest that there is a nanobody-nanobody interface. Does maybe formation of this interface "solidify" what the authors call "the perch" of the nanobody?

4) Please explain why two different expression systems (293 cells and Sf9 cells) were used for the different mutants. Is there any concern about different post-translational modification of the Kv1.3 protein?

5) Please explain why a molar ratio of 3:1 of nanobody to channel protein was used for the cryo-EM sample preparation. Why 3:1 if the nanobody is bound with a 4:1 ratio?

Reviewer #2

(Remarks to the Author)
The manuscript by Selvakumar et al. reports an elegant study that combines functional and structural approaches to

elucidate the mechanism responsible for the inhibition of the potassium channel Kv1.3 channel by a specific nanobody. It emerged from a previous study published by the Swartz lab, which demonstrated that the nanobody NB1.3 promotes C-type inactivation in Kv1.3 and reported the cryo-EM structure of the Kv1.3-NB1.3 complex. In the study under review, the authors delved deeper into the question of how NB1.3 promotes C-type inactivation and, in doing so, gained new insights into the structural basis of Kv channel C-type inactivation and the interactions that stabilize the conducting conformation of the ion selectivity filter.

This effort represents a tour de force, which solved and carefully examined a total of seven high-resolution cryo-EM structures, including wild-type and three mutants (sometimes in both the apo and NB1.3-bound conformations). All mutations were also characterized under voltage clamping conditions to infer the functional significance of possible interactions and the mechanisms. The authors concluded that NB1.3 interacts with the extracellular S1-S2 linker and the turret region, with the latter having a greater impact on the interaction and structural effects of NB1.3 binding. The authors provide convincing evidence to suggest that a previously unknown network of hydrophobic interactions (involving F428 in the turret, M450 in the pore helix and W436 in the selectivity filter) helps determine the stability of the ion selectivity filter conducting conformation. Moreover, the cryo-EM structures suggest that binding of NB1.3 causes a modest turret rearrangement that is sufficient to disrupt the hydrophobic network and thereby facilitate and stabilize the dilation of the narrow pore that is responsible for C-type inactivation.
Overall, this well-written manuscript reports compelling and impactful results with clear mechanistic implications. The comments presented below may help clarify the methodology and the results and strengthen the conclusions.

Major Comments

1) To demonstrate the importance of F428 in the turret, the authors perturb this site by testing the functional effects of three mutations under voltage clamping conditions: F428A, F428I and F428L. F428A most significantly promoted fast and profound C-type inactivation. This result helped suggest the novel importance of F428 in the hydrophobic network that stabilizes the conducting conformation of the selectivity filter, which is key for the proposed mechanism. It is, however, not clear why the authors did not pursue cryo-EM characterization of Kv1.3/F428A. Considering that the authors were able to solve seven cryo-EM structures in this study (including the wild type Kv1.3 and several mutants), what hurdle(s) prevented cryo-EM characterization of Kv1.3/F428A? This structure would have probably revealed a novel conformation and strengthen the conclusions, especially with respect to the key role of F428 and the turret in C-type inactivation and the mechanism of action of NB1.3.

2) On Fig. 7d, the authors show the superimposed turret structures of key mutants and the wild type in the absence and presence of NB1.3. The rearrangements are, however, not limited to the turret. It may benefit the readers' appreciation of the results and conclusions if the authors were to include overlays of the entire pore domains, especially for the following cases: 1) Wild type Kv1.3 -/+ NB1.3; 2) H451V -/+ NB1.3; 3) G427H -/+ NB1.3. If space is limited, presenting these overlays as Extended Data would be sufficient.

Minor Comments

1) Under Methods, the authors indicate that the best first-order Boltzmann fit was used to describe the G/V curves. However, the G/V data presented are not accompanied by this analysis, including best-fit parameters.

2) Under Methods, the authors indicate that the best single or double exponential fit was used to describe the trajectories of macroscopic inactivation. Also, under Results, the authors state that Kv1.3 macroscopic inactivation is best described by a double exponential function. However, the graphs in Fig. 1h, 3c and 6b only report one time constant. Which time constant is plotted? Is this the weighted average of the fast and slow time constants?

3) To characterize the inhibition of Kv1.3 currents by NB1.3 (e.g., Fig. 1g), the authors use a steady-state inactivation protocol. It is thus apparent that because NB1.3 binding stabilizes the inactivated state of wild type Kv1.3 the steady state inactivation curve is leftward shifted. However, the H451V and G427H mutants do not exhibit this shift. Instead, even in the absence of NB1.3, the curves exhibit a U-shape possibly because some inactivation may occur from a pre-open closed state and the mutation-induced stabilization of the conducting conformation. Although delving into these observations is probably beyond the scope of this study, I suggest to descriptively conceptualize the inhibitory effect of NB1.3 in terms of the hyperpolarizing shift (or absence thereof) as it is related to the mechanism of action. Also, it should be noted that NB1.3 "potentiates" the current expressed by H451V and G427H at the most depolarized pre-pulse voltages of the steady state inactivation curve. It is, however, not clear why.

4) As pointed out above, NB1.3 stabilizes the C-type inactivated state and, therefore, the steady-state inactivation curve shifts to the left. In addition, NB1.3 greatly reduces the Gmax (Fig. 1j). These observations deserve more attention because H451V and P424G eliminate the leftward shift, and yet, they still induce a substantial inhibition of the Gmax. In contrast, G427H eliminates both the leftward shift and the inhibition of the Gmax. In terms of the proposed mechanism, it is not clear why H451V and G427H produce these differences. The authors appear to underplay the significance of these observations in the final paragraph of the discussion.

5) Typo on line 400. I believe the authors meant "F428-M450-W436 network".

Reviewer #3

(Remarks to the Author)
In this manuscript, Selvakumar et al characterize in detail the interaction between the Kv1.3 channel and the nanobody NB1.3. The manuscript can be seen as a continuation of their 2022 paper (cited as reference 5 in the manuscript) in which they had already determined a cryo-EM structure of NB1.3 bound to Kv1.3 in an inactivated conformation. Now, they investigate how exactly NB1.3 induces C-type inactivation of Kv1.3. For this, they determine the first structures of Kv1.3 in a conductive conformation, which they then use as starting point to determine further structures in complex with NB1.3. Together with mutational analysis and electrophysiology measurements, they provide an extensive and thorough characterization of binding interfaces and their contribution to C-type inactivation induced by NB1.3.

Overall, the data presented in the manuscript are of high quality and experiments are well though through. However, there are some points the authors should clarify before publication:

1. A major concern is that the manuscript is written mostly for Kv1.3 experts and this makes it a bit inaccessible for non-experts. In order to make it interesting and digestible for the broad readership of Nature Comms, both the text and the figures should be modified in order to allow easier and more intuitive understanding.
For example, the beginning of the results section should be rewritten to better introduce the different conformations shown in Fig. 1a-c and the structural rearrangements expected during C-type inactivation. Also, an overview of the whole structure of Kv1.3 would be good to have in Fig. 1 in order to help non-experts to understand the architecture of Kv1.3 and which part of it is shown in Fig. 1a-d,k or also Fig. 1l. Furthermore, it would be very useful to have an (Extended Data) Figure with structural overviews of all their structures (reconstructions and models) side by side to better appreciate claims made in the manuscript regarding overall structural similarity, different conformations of the NB1.3 binding etc.

2. In l. 132, the authors claim that the apparent affinity of NB1.3 is not affected by the H451V mutant. This is a bit misleading as they only show experiments with two concentrations, 100 nM and 1 uM, which are both a lot higher compared to the described low nM dissociation constant of NB1.3. Thus, the authors should make it clear that they cannot make claims about a potential influence of the H451 mutation on the affinity, but even if there is, it does not affect the interaction at the (saturating) concentrations used in their experiments. The same also applies to l. 183 and l. 275.

3. In the sentence starting in l. 163, they write that the Kv1.3-NB1.3 interfaces are very similar in all three structures (the two new conformations and their previous structure). It would be good to show this in a figure, e.g. by superpositions.

4. The authors describe the two conformations they obtained of the Kv1.3-NB1.3 complex only very briefly and do not comment further on these two different conformations. Do they think there is any functional relevance in these two distinct conformations, or do they just represent two snapshots in a partially flexible arrangement?

5. It would be helpful to have structural superpositions andor morph movies of the different conformations they see in the selectivity filter and the interaction networks to supplement e.g. Fig. 4.

6. The manuscript provides a really extremely detailed analysis of interaction networks that change during Kv1.3 inactivation. However, in my opinion, in between these very lengthy and detailed descriptions, I am missing a bit the overall message. Also in the Discussion, the authors focus mainly on specific interactions between their nanobody and the channel, which in my opinion should not b the main point. In the end, the nanobody is just a tool to identify residues important for C-type interaction and should thus not be so much in the center. Instead, the authors should rather focus more on their model of C-type inactivation and the involved structural rearrangements, as this is the biologically relevant portion.

7. The claim in l. 403 that the results presented here could be used as a foundation for NB regulation of other channels is very speculative since C-type inactivation has been demonstrated for only a subset of voltage-gated channels, and these channels also diverge in the architecture of the pore domain, the turret and the S1-S2 loop. The authors should remove this claim or only mention channels that are sufficiently close to Kv1.3 in the relevant structural features.

Tobias Raisch
Max Planck Institute of Molecular Physiology, Dortmund, Germany

Version 1:

Reviewer comments:

Reviewer #1

(Remarks to the Author)
The authors have addressed all my previous concerns.

Heike Wulff

Reviewer #2

(Remarks to the Author)
The authors have addressed my comments satisfactorily.

Reviewer #3

(Remarks to the Author)
The authors have answered most of the points I have raised in a satisfactory manner. In particular, I appreciate the effort of preparing new figures such as Fig.1 that will help non-experts to understand more easily what the findings are about.

The only thing that still bothers me a bit is the Discussion which, in my opinion, is still too much about small details and too little about the big picture. I do get the point the authors make in reply to my original remark, i.e. that it is necessary to understand in miniscule detail the interactions of the nanobody with Kv1.3 in order to optimize NB1.3 further and design nanobodies against other channels. However, I fear that this point does not get across sufficiently in the Discussion as it is more a little-by-little description of effects of Kv1.3 residues and mutations instead of pointing out what structural features such improved nanobodies should have. If this study is to be the basis for nanobody optimization and design, the authors could e.g. make suggestions for mutations in NB1.3 to increase its affinity towards the inactivated conformation of Kv1.3, or to switch its specificity in order to target other channels.

Open Access This Peer Review File is licensed under a Creative Commons Attribution 4.0 International License, which permits use, sharing, adaptation, distribution and reproduction in any medium or format, as long as you give appropriate credit to the original author(s) and the source, provide a link to the Creative Commons license, and indicate if changes were made.
In cases where reviewers are anonymous, credit should be given to 'Anonymous Referee' and the source.
The images or other third party material in this Peer Review File are included in the article's Creative Commons license, unless indicated otherwise in a credit line to the material. If material is not included in the article's Creative Commons license and your intended use is not permitted by statutory regulation or exceeds the permitted use, you will need to obtain permission directly from the copyright holder.
To view a copy of this license, visit https://creativecommons.org/licenses/by/4.0/

NCOMMS-25-77427

Nanobody regulation of C-type inactivation in Kv1.3 channels

Purushotham Selvakumar, Kenton J. Swartz and Ana I. Fernández-Mariño

We greatly appreciate the thoughtful guidance of the reviewers. We have now prepared a revised version for further consideration. The comments of the reviewers are shown below in black text and our responses and descriptions of the changes made are shown in blue text.

REVIEWER COMMENTS

Reviewer #1 (Remarks to the Author):

The authors of this study previously reported the cryo-EM structures of Kv1.3 in the apo-state and in complex with the NB1.3 nanobody and an ShK-knotbody. However, the previously resolved apo structure was in the C-type inactivated state with a dilated selectivity filter and the field is therefore missing structures of open, conducting states of Kv1.3 and insight into the mechanism of action of the NB1.3 nanobody, which is binding to the turret of the channel and inhibits the channel by speeding up inactivation.

In the current study the authors use a combination of mutagenesis, electrophysiology and cryo-EM to characterize and then capture several mutants with reduced C-type inactivation (H451V, G427H) in the open conducting state with a fully occupied selectivity filter. The authors further map the interface between the nanobody and the channel turret and the S1-S2 loop and again obtain the structure of a key mutant (P424G) to obtain deeper insight into how the nanobody induces C-type inactivation by modulating the turret and eventually dilating the selectivity filter through modification of a network of hydrophobic residues.

The work is important in providing insights into the mechanisms of C-type inactivation in Kv1 family channels and possibly enabling design of nanobodies targeting tetrameric cation channels.

We appreciate the supportive comments of this reviewer and their thoughtful suggestions.

As a reviewer I have several suggestions for the authors:

1) Would you dare to make a little movie of morphed structures showing how the nanobody induces C-type inactivation? If you do not think this is possible at this point, please rebut.

This is a good suggestion. We prepared a movie (Movie S1) where we morph between H451V to WT with NB in D1 and then D3 inactivated states.

2) Please clarify if the two nanobody conformations observed with the H451V mutant with nanobodies either leaning inward or outward were also observed in the previously captured structure of the nanobody with the WT channels. Fig 2 seems to suggest that the previous structure only showed the inward conformation. Please specify the confirmation and maybe speculate if the nanobody progresses from outward to inward when it inhibits the WT channel.

Our earlier structure of NB1.3 bound to WT Kv1.3 showed the NB adopting the outward leaning conformation (all 123,722 particles). In the current dataset with H451V plus NB1.3, 115,627 particles were inward leaning and 109,882 were outward leaning. We do not have any evidence to suggest these two conformations are related to the mechanism of action of the NB because the K76A mutant of the NB that should destabilize the inward leaning conformation has similar activity to the WT NB. We also only see the outward leaning conformation in the G427H mutant (259,382 particles) that disfavors inactivation, suggesting that the two conformations seen in H451V are not simply related to the mutant interfering with inactivation. We think this wobble is intriguing, and we have expanded the presentation of these observations in the results section to provide more information to readers, but we are not sure there is anything to speculate about at this point.

3) Please also specify if there are any direct contacts between the four nanobodies in the inward confirmation. Again, Figure 2 seems to suggest that there is a nanobody-nanobody interface. Does maybe formation of this interface "solidify" what the authors call "the perch" of the nanobody?

The only contact between adjacent NBs in the inward leaning conformation is the interaction between K76 and S71 (see Figure S4k) and the K76A mutant has no discernable effect on the NB. As noted above, we have expanded our discussion of these two conformations, but we do not understand how they are related to the mechanism by which the NB promotes inactivation.

4) Please explain why two different expression systems (293 cells and Sf9 cells) were used for the

different mutants. Is there any concern about different post-translational modification of the Kv1.3 protein?

We used EXPI293 and tsA201 cells for expression and both cell types are commonly used expression systems that we and others use interchangeably. P424G and H451V were expressed in EXPI 293 cells with direct transfection of DNA into the cells whereas in the case of G427H, we used Bac Mam expression where P1 and P2 viruses were produced in SF9 cells and tsA201 cells were transfected using P2 virus. EXPI 293 and tsA201 cells are very similar, and both are standard methods for production of membrane proteins for structure determination.

5) Please explain why a molar ratio of 3:1 of nanobody to channel protein was used for the cryo-EM sample preparation. Why 3:1 if the nanobody is bound with a 4:1 ratio?

Kv1.3 is a homotetrameric protein and each individual subunit binds one NB1.3. We used a 3:1 ratio of molar excess of NB1.3 to each Kv1.3 subunit (revised methods, lines 706 and 708), meaning that for each Kv1.3 subunit there is 3-fold excess of NB1.3. We have modified this section to be clearer.

Reviewer #2 (Remarks to the Author):

The manuscript by Selvakumar et al. reports an elegant study that combines functional and structural approaches to elucidate the mechanism responsible for the inhibition of the potassium channel Kv1.3 channel by a specific nanobody. It emerged from a previous study published by the Swartz lab, which demonstrated that the nanobody NB1.3 promotes C-type inactivation in Kv1.3 and reported the cryo-EM structure of the Kv1.3-NB1.3 complex. In the study under review, the authors delved deeper into the question of how NB1.3 promotes C-type inactivation and, in doing so, gained new insights into the structural basis of Kv channel C-type inactivation and the interactions that stabilize the conducting conformation of the ion selectivity filter.

This effort represents a tour de force, which solved and carefully examined a total of seven high-resolution cryo-EM structures, including wild-type and three mutants (sometimes in both the apo and NB1.3-bound conformations). All mutations were also characterized under voltage clamping conditions to infer the functional significance of possible interactions and the mechanisms. The authors concluded that NB1.3 interacts with the extracellular S1-S2 linker and the turret region, with the latter having a greater impact on the interaction and structural effects of NB1.3 binding. The authors provide convincing evidence to suggest that a previously unknown network of

hydrophobic interactions (involving F428 in the turret, M450 in the pore helix and W436 in the selectivity filter) helps determine the stability of the ion selectivity filter conducting conformation. Moreover, the cryo-EM structures suggest that binding of NB1.3 causes a modest turret rearrangement that is sufficient to disrupt the hydrophobic network and thereby facilitate and stabilize the dilation of the narrow pore that is responsible for C-type inactivation. Overall, this well-written manuscript reports compelling and impactful results with clear mechanistic implications. The comments presented below may help clarify the methodology and the results and strengthen the conclusions.

We appreciate the supportive comments of this reviewer and their thoughtful suggestions.

Major Comments

1) To demonstrate the importance of F428 in the turret, the authors perturb this site by testing the functional effects of three mutations under voltage clamping conditions: F428A, F428I and F428L. F428A most significantly promoted fast and profound C-type inactivation. This result helped suggest the novel importance of F428 in the hydrophobic network that stabilizes the conducting conformation of the selectivity filter, which is key for the proposed mechanism. It is, however, not clear why the authors did not pursue cryo-EM characterization of Kv1.3/F428A. Considering that the authors were able to solve seven cryo-EM structures in this study (including the wild type Kv1.3 and several mutants), what hurdle(s) prevented cryo-EM characterization of Kv1.3/F428A? This structure would have probably revealed a novel conformation and strengthened the conclusions, especially with respect to the key role of F428 and the turret in C-type inactivation and the mechanism of action of NB1.3.

This is an interesting suggestion and one that we considered undertaking. However, the F428A mutant does not express well enough to obtain sufficient quantities for structure determination. In our functional experiments, we had to use high RNA concentrations to measure even relatively small currents in voltage-clamp experiments.

2) On Fig. 7d, the authors show the superimposed turret structures of key mutants and the wild type in the absence and presence of NB1.3. The rearrangements are, however, not limited to the turret. It may benefit the readers' appreciation of the results and conclusions if the authors were to include overlays of the entire pore domains, especially for the following cases: 1) Wild type Kv1.3-/+ NB1.3; 2) H451V-/+ NB1.3; 3) G427H-/+ NB1.3. If space is limited, presenting these overlays as Extended Data would be sufficient.

We have added a new supplementary figure (Figure S10) showing the requested overlays of the pore domains for 1) WT Kv1.3-/+ NB1.3, 2) H451V-/+ NB1.3 and 3) G427H-/+ NB1.3.

Minor Comments

1) Under Methods, the authors indicate that the best first-order Boltzmann fit was used to describe the G/V curves. However, the G/V data presented are not accompanied by this analysis, including best-fit parameters.

We have now added fits of the Boltzmann equation for all of the G-V and steady-state inactivation relations.

2) Under Methods, the authors indicate that the best single or double exponential fit was used to describe the trajectories of macroscopic inactivation. Also, under Results, the authors state that Kv1.3 macroscopic inactivation is best described by a double exponential function. However, the graphs in Fig. 1h, 3c and 6b only report one time constant. Which time constant is plotted? Is this the weighted average of the fast and slow time constants?

As the reviewer points out, inactivation in the WT Kv1.3 channel is indeed best described by fitting a double exponential function to current traces, in particular if they are longer than 1 sec. For all data presented in the current manuscript, τ values for inactivation were obtained by fitting a single exponential function to current traces because these are sufficient to describe the effects of the NB and of most mutations. The one exception is P424G, where the mutation specifically speeds inactivation by removing the slow component observed in double exponential fits for the WT channel. For that reason, we compare data for P424G to double exponential fits to the WT channel that we reported in our earlier study (ref. 5). We now clarify this in the methods section and figure legends so there is hopefully no confusion.

3) To characterize the inhibition of Kv1.3 currents by NB1.3 (e.g., Fig. 1g), the authors use a steady-state inactivation protocol. It is thus apparent that because NB1.3 binding stabilizes the inactivated state of wild type Kv1.3 the steady state inactivation curve is leftward shifted. However, the H451V and G427H mutants do not exhibit this shift. Instead, even in the absence of NB1.3, the curves exhibit a U-shape possibly because some inactivation may occur from a pre-open closed state and the mutation-induced stabilization of the conducting conformation. Although delving into these observations is probably beyond the scope of this study, I suggest to descriptively conceptualize the inhibitory effect of NB1.3 in terms of the hyperpolarizing shift (or absence thereof) as it is related to the mechanism of action. Also, it should be noted that NB1.3 “potentiates” the current expressed by H451V and G427H at the most depolarized pre-pulse voltages of the steady state inactivation curve. It is, however, not clear why.

This is an excellent point, and we now conceptualize the effects of the NB on inactivation using the shifts in stead-state inactivation relations. We have also added additional comments in the results and discussion sections about effects of the NB that appear to be unrelated to inactivation, such as stabilizing the open state when inactivation is disfavored by mutations.

4) As pointed out above, NB1.3 stabilizes the C-type inactivated state and, therefore, the steady-state inactivation curve shifts to the left. In addition, NB1.3 greatly reduces the Gmax (Fig. 1j). These observations deserve more attention because H451V and P424G eliminate the leftward shift, and yet, they still induce a substantial inhibition of the Gmax. In contrast, G427H eliminates both the leftward shift and the inhibition of the Gmax. In terms of the proposed mechanism, it is not clear why H451V and G427H produce these differences. The authors appear to underplay the significance of these observations in the final paragraph of the discussion.

This is also an excellent point. We have added additional text in the results and discussion sections pointing out effects of the NB that appear to be unrelated to its effects on inactivation. We currently do not understand all of the effects of the NB on aspects of gating unrelated to inactivation, but the point we were trying to make in the discussion was that our results clearly indicate that a NB binding to both the VSD and PD can influence many aspects of gating, motivating the development of NBs against many other tetrameric cation channels.

5) Typo on line 400. I believe the authors meant “F428-M450-W436 network”.

Thank you for catching this! The error has been corrected.

Reviewer #3 (Remarks to the Author):

In this manuscript, Selvakumar et al characterize in detail the interaction between the Kv1.3 channel and the nanobody NB1.3. The manuscript can be seen as a continuation of their 2022 paper (cited as reference 5 in the manuscript) in which they had already determined a cryo-EM structure of NB1.3 bound to Kv1.3 in an inactivated conformation. Now, they investigate how exactly NB1.3 induces C-type inactivation of Kv1.3. For this, they determine the first structures of Kv1.3 in a conductive conformation, which they then use as starting point to determine further structures in complex with NB1.3. Together with mutational analysis and electrophysiology measurements, they provide an extensive and thorough characterization of binding interfaces and their contribution to C-type inactivation induced by NB1.3.

Overall, the data presented in the manuscript are of high quality and experiments are well though through. However, there are some points the authors should clarify before publication:

We thank the reviewer for these supportive comments and thoughtful suggestions for revision.

1. A major concern is that the manuscript is written mostly for Kv1.3 experts and this makes it a bit inaccessible for non-experts. In order to make it interesting and digestible for the broad readership of Nature Comms, both the text and the figures should be modified in order to allow easier and more intuitive understanding.
For example, the beginning of the results section should be rewritten to better introduce the different conformations shown in Fig. 1a-c and the structural rearrangements expected during C-type inactivation. Also, an overview of the whole structure of Kv1.3 would be good to have in Fig. 1 in order to help non-experts to understand the architecture of Kv1.3 and which part of it is shown in Fig. 1a-d,k or also Fig. 1l. Furthermore, it would be very useful to have an (Extended Data) Figure with structural overviews of all their structures (reconstructions and models) side by side to better appreciate claims made in the manuscript regarding overall structural similarity, different conformations of the NB1.3 binding etc.

These are all wonderful ideas, and we have systemically gone through the text to increase accessibility and added many of the suggested changes to figures. Specifically, we added a new Figure 1 to introduce the Kv1.3 channel and background information on conducting and inactivated conformations of the selectivity filter from recent related work on the Shaker Kv channel. We have also added a new supplementary figure (Figure S11) showing cryo-EM maps and models for the new structures reported here.

2. In l. 132, the authors claim that the apparent affinity of NB1.3 is not affected by the H451V mutant. This is a bit misleading as they only show experiments with two concentrations, 100 nM and 1 uM, which are both a lot higher compared to the described low nM dissociation constant of NB1.3. Thus, the authors should make it clear that they cannot make claims about a potential influence of the H451 mutation on the affinity, but even if there is, it does not affect the interaction at the (saturating) concentrations used in their experiments. The same also applies to l. 183 and l. 275.

Another very thoughtful point. We have revised these areas of the text to be more precise as suggested by the reviewer.

3. In the sentence starting in l. 163, they write that the Kv1.3-NB1.3 interfaces are very similar in

all three structures (the two new conformations and their previous structure). It would be good to show this in a figure, e.g. by superpositions.

We have added a new supplementary figure (Figure S9) where we superimpose the three new structures of mutant Kv1.3 channels with NB1.3 bound along with the earlier structure of WT Kv1.3 with NB1.3 bound.

4. The authors describe the two conformations they obtained of the Kv1.3-NB1.3 complex only very briefly and do not comment further on these two different conformations. Do they think there is any functional relevance in these two distinct conformations, or do they just represent two snapshots in a partially flexible arrangement?

We think the two conformations likely represent two snapshots in a partially flexible arrangement. Our earlier structure of NB1.3 bound to WT Kv1.3 showed the NB adopting the outward leaning conformation (all 123,722 particles). In the current dataset with H451V plus NB1.3, 115,627 particles were inward leaning and 109,882 were outward leaning. We do not have any evidence to suggest these two conformations are related to the mechanism of action of the NB because the K76A mutant of the NB that should destabilize the inward leaning conformation has similar activity to the WT NB. We also only see the outward leaning conformation in the G427H mutant (259,382 particles) that disfavors inactivation, suggesting that the two conformations seen in H451V are not simply related to the mutant interfering with inactivation. We think this wobble is intriguing, and we have expanded the presentation of these observations in several places to give the reader a clearer picture.

5. It would be helpful to have structural superpositions and or morph movies of the different conformations they see in the selectivity filter and the interaction networks to supplement e.g. Fig. 4.

We have added superimposed structures of the PDs for all the available structures in a new supplementary figure (Figure S10) and we have added a movie (Movie S1) showing a morph between H451V in a conducting state to D1 and then D3 with NB1.3 bound to WT Kv1.3.

6. The manuscript provides a really extremely detailed analysis of interaction networks that change during Kv1.3 inactivation. However, in my opinion, in between these very lengthy and detailed descriptions, I am missing a bit the overall message. Also in the Discussion, the authors focus mainly on specific interactions between their nanobody and the channel, which in my

opinion should not b the main point. In the end, the nanobody is just a tool to identify residues important for C-type interaction and should thus not be so much in the center. Instead, the authors should rather focus more on their model of C-type inactivation and the involved structural rearrangements, as this is the biologically relevant portion.

This is a very helpful comment, and we have tried to make the key take home messages clearer in the revised manuscript. We agree that NB1.3 is a useful tool to understand regions that regulate C-type inactivation, and as such this provides new information about C-type inactivation. However, we also see these NBs as potential therapeutics and that elucidating their mechanisms of action will provide valuable information for optimizing their therapeutic potential. Finally, we think what we have learned here provides motivation for developing new NBs against other tetrameric cation channels that have a similar architecture to Kv1.3 because we show how NB1.3 interacting with both the VSD and the PD can modulate inactivation and other aspects of gating (that we have not yet mechanistically explored). We have systemically gone through the text to hopefully make the key take home messages clearer.

7. The claim in l. 403 that the results presented here could be used as a foundation for NB regulation of other channels is very speculative since C-type inactivation has been demonstrated for only a subset of voltage-gated channels, and these channels also diverge in the architecture of the pore domain, the turret and the S1-S2 loop. The authors should remove this claim or only mention channels that are sufficiently close to Kv1.3 in the relevant structural features.

We have rephrased this to hopefully make our point more clearly. We did not intend to claim that the specific interactions between NB1.3 and Kv1.3 will generalize to other tetrameric channels. Indeed, we already know that NB1.3 does not modulate the activity of most other Kv1 channel subtypes. Our intent is as stated in response to the previous comment that the demonstration that NB1.3 binds to both the VSD and PD of Kv1.3 and modulates not only inactivation, but other aspects of gating, makes us optimistic that generating new NBs against other channels with a similar architecture will yield valuable new modulators of those channels.
